# Fc gamma receptors: Their evolution, genomic architecture, genetic variation, and impact on human disease

**DOI:** 10.1111/imr.13401

**Published:** 2024-09-30

**Authors:** Sarah Frampton, Rosanna Smith, Lili Ferson, Jane Gibson, Edward J. Hollox, Mark S. Cragg, Jonathan C. Strefford

**Affiliations:** ^1^ Cancer Genomics Group, Faculty of Medicine, School of Cancer Sciences University of Southampton Southampton UK; ^2^ Antibody and Vaccine Group, Faculty of Medicine, School of Cancer Sciences, Centre for Cancer Immunology University of Southampton Southampton UK; ^3^ Department of Genetics, Genomics and Cancer Sciences College of Life Sciences, University of Leicester Leicester UK

**Keywords:** antibodies, evolution, Fc receptors, genetics, immunotherapy

## Abstract

Fc gamma receptors (FcγRs) are a family of receptors that bind IgG antibodies and interface at the junction of humoral and innate immunity. Precise regulation of receptor expression provides the necessary balance to achieve healthy immune homeostasis by establishing an appropriate immune threshold to limit autoimmunity but respond effectively to infection. The underlying genetics of the *FCGR* gene family are central to achieving this immune threshold by regulating affinity for IgG, signaling efficacy, and receptor expression. The *FCGR* gene locus was duplicated during evolution, retaining very high homology and resulting in a genomic region that is technically difficult to study. Here, we review the recent evolution of the gene family in mammals, its complexity and variation through copy number variation and single‐nucleotide polymorphism, and impact of these on disease incidence, resolution, and therapeutic antibody efficacy. We also discuss the progress and limitations of current approaches to study the region and emphasize how new genomics technologies will likely resolve much of the current confusion in the field. This will lead to definitive conclusions on the impact of genetic variation within the *FCGR* gene locus on immune function and disease.

## 1 FUNCTIONAL IMPORTANCE OF FcγRs

The Fc gamma receptors (FcγRs) are a family of cell‐surface proteins that are expressed on a variety of lymphoid, myeloid, and non‐immune cells, which function by binding the Fc portion of immunoglobulin G (IgG). They play a pivotal role in orchestrating both the humoral and innate immune response, serving as an immunological rheostat to achieve the delicate balance between facilitating appropriate responses to infections and averting autoimmunity.^1^ The FcγRs also have a crucial role in determining the efficacy of monoclonal antibody (mAb) immunotherapy^2^, ^3^ and are implicated in the genetic predisposition to autoimmune, infectious, and inflammatory diseases.

**FIGURE 1:**
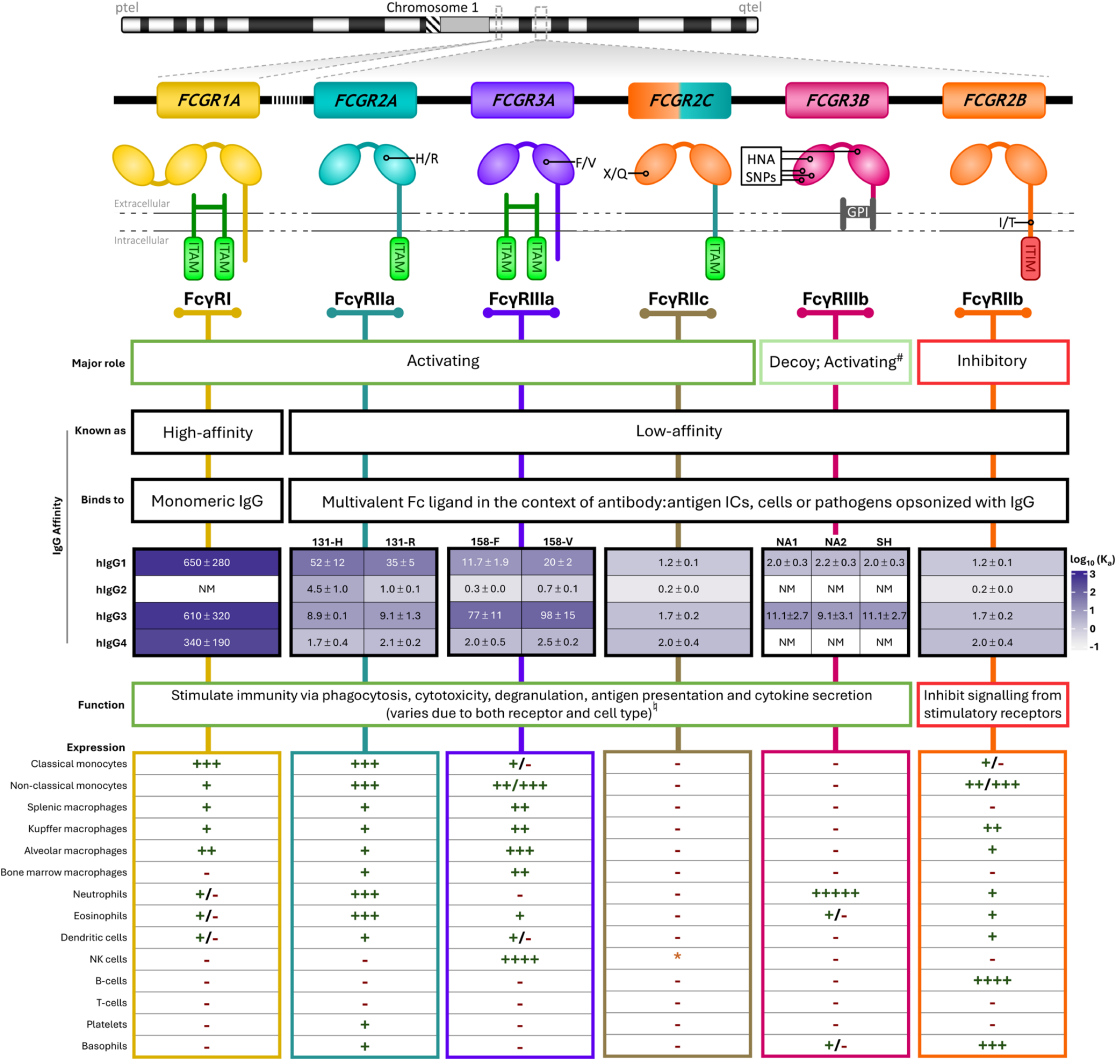
Function, IgG affinity, and expression of the six human Fcγ receptors (FcγRs). FcγRs bind to the Fc region of immunoglobulin G (IgG) and help coordinate the humoral immune response. FcγRI, FcγRIIa, FcγRIIc, and FcγRIIIa signal for cell activation through an immunoreceptor tyrosine‐based activation motif (ITAM), located in the cytoplasmic tail of the receptor (FcγRIIa, FcγRIIc), or the noncovalently associated FcR gamma chain (FcγRI, FcγRIIIA). In NK cells, FcγRIIIA can also be associated with the CD3 ζ‐chain.^11^, ^12^, ^13^ The aggregation of activating FcγRs at the cell surface facilitates the phosphorylation of tyrosine residues in the ITAMs by Src family protein tyrosine kinases (PTKs), triggering a series of downstream signaling cascades leading to cellular activation.^14^ FcγRIIb is the sole inhibitory receptor and has an immunoreceptor tyrosine‐based inhibition motif (ITIM). Phosphorylation of the ITIM tyrosine residues by Src family PTKs during aggregation of the inhibitory FcγRIIb mediates binding of several phosphatases that then proceed to dephosphorylate other proteins and lipids, terminating activating signaling, resulting in cellular inhibition.^8^ This functions to limit responses through the activating FcγRs as well as other stimulatory receptors, like the B‐cell receptor. FcγRIIb can also reduce activity by competing for activating FcγRs for binding to IgG.^9^ FcγRIIIb lacks both transmembrane and cytoplasmic domains, with membrane attachment occurring through a glycosylphosphatidylinositol (GPI) anchor instead. ^#^While sometimes labeled a decoy receptor due to the lack of intracellular domains and direct signaling capacity, FcγRIIIb has demonstrated the capability for intracellular signaling transduction and cell activation through association with other cell‐surface receptors, such as FcγRIIa^15^, ^16^, ^17^, ^18^, ^19^ and integrins.^20^ Only FcγRI is considered high‐affinity as it can engage with monomeric IgG, whereas the remaining FcγRs interact solely with multimeric IgG immune complexes (ICs) or opsonized targets and thus are regarded as low‐affinity. Binding affinities for the different human IgG (hIgG) subclasses have been determined using surface plasmon resonance (SPR). Shown above are affinity constants (×10^5^ M^−1^) for K_A_ with standard deviation.^6^ Most effector leukocytes express a combination of activating and inhibitory FcγRs that dictate the induction of IgG‐mediated immune responses through their opposing signaling capacities. The responsiveness of an effector cell to IgG is also influenced by differentiation state and inflammatory stimuli as these regulate FcγR expression. ♮There is uncertainty around the impact and functional capacity of FcγRIIc, with its previous designation as a pseudogene^21^ and expression controlled by the presence or absence of a polymorphism dictating a stop codon. Expression of FcγRs has been measured by various methods in peripheral effector cells,^22^, ^23^ tissue‐resident macrophages,^24^ and dendritic cells.^7^ “+” represents constitutive expression (+<++<+++<++++<+++++), “‐” represents absence of expression, “+/−” represents inducible expression, and * represents that expression is determined by *FCGR2C* genotype.

Characterized by their intracellular signaling capabilities and affinity for IgG, the six human FcγRs are FcγRI (CD64), FcγRIIa (CD32A), FcγRIIb (CD32B), FcγRIIc (CD32C), FcγRIIIa (CD16A), and FcγRIIIb (CD16B). FcγRI is the sole high‐affinity receptor, whereas FcγRIIa, FcγRIIb, FcγRIIc, FcγRIIIa, and FcγRIIIb are classified as low‐affinity receptors.^4^ While both high‐ and low‐affinity FcγRs bind antibody immune complexes (ICs) with high avidity, only the high‐affinity FcγRI stably binds monomeric IgG. In contrast, low‐affinity receptors bind to multivalent Fc regions on IgG‐opsonized cells, pathogens, and within soluble ICs.^2^, ^5^, ^6^ The majority of FcγRs trigger intracellular signaling pathways that elicit immune effector functions to eliminate opsonized targets,^7^ and are so termed activating FcγRs. In contrast, the sole inhibitory FcγR, FcγRIIb, can suppress the effects of activating receptors by triggering an inhibitory signaling cascade, and competing for ligand binding at the cell surface.^8^, ^9^ The simultaneous expression of both activating and inhibitory FcγRs on a single cell facilitates precise modulation of the stimulation threshold, referred to as the activating/inhibitory (A/I) ratio.^10^ Depending on the specific FcγR and cell type, diverse cellular responses are elicited upon reaching the activation threshold, including phagocytosis, cytokine secretion, reactive oxygen burst, and cytotoxic granule release.^5^ Consequently, in conjunction with the varying affinities of different IgG isotypes for various FcγR and cell context‐dependent intracellular signaling pathways, the tight regulation of FcγR expression and activation ensures an appropriate and coordinated immune response. Affinity, expression profiles, and activities of the FcγRs are summarized in Figure 1, alongside details of how they elicit their downstream effects. A central component of FcγR regulation is the germline‐encoded variation in the *FCGR* genes, which themselves have arisen during evolution as described below.

## 2 EVOLUTION OF *FCGRs*

The *FCGR* gene family arose in our common ancestor with bony fish during the development of the adaptive immune system, coinciding with the emergence of their IgG ligands.^25^, ^26^ Investigating different *FCGR* loci has highlighted how the FcγR functional repertoire has evolved through multiple events of duplication and divergence, enabling adaptation to immunological challenges.

### 2.1 High‐affinity *FCGRs*

The human FcγRI receptor is encoded by the *FCGR1A* gene, located on chromosome 1q21.2^27^ and separate from the low‐affinity locus. The high‐affinity FcγRI is unique as it exhibits a third extracellular Ig‐like domain,^28^ distinguishing it from the low‐affinity FcγRs which only have two, that facilitates binding to both monomeric and aggregated IgG.^6^, ^29^ Additionally, there are two pseudogenes, *FCGR1B* on chromosome 1p11.2 and *FCGR1C* on chromosome 1q21.1,^30^ that are not believed to contribute to the surface expression of FcγRI.^31^ The evolution of the three *FCGR1* genes is thought to have involved duplication of an ancestral gene, likely located on chromosome 1p,^32^ producing *FCGR1A* and a second homolog with a 6 base‐pair (bp) insertion. Further duplication of this gene likely resulted in a third *FCGR1* gene. The separation of *FCGR1A* and *FCGR1C* from *FCGR1B* onto the long arm of chromosome 1 is hypothesized to have occurred following human divergence from chimpanzees through a pericentric inversion event.^30^, ^33^

The origins of the high‐affinity *FCGR* genes are less understood than those of the low‐affinity *FCGR* locus; in particular, the exact point of divergence is yet to be classified. One study has reported the low‐affinity receptors emerged first in mammalian evolution.^25^ This was postulated from analysis of the opossum genome, diverging from placental mammals 130 million years ago, as two‐domain FcγR genes were present, yet three‐domain receptors were absent. However, this may be attributed to technical limitations of genome assembly or evolutionary loss in the opossum lineage rather than the actual absence of the receptor.^26^

### 2.2 Low‐affinity *FCGRs*

**FIGURE 2:**
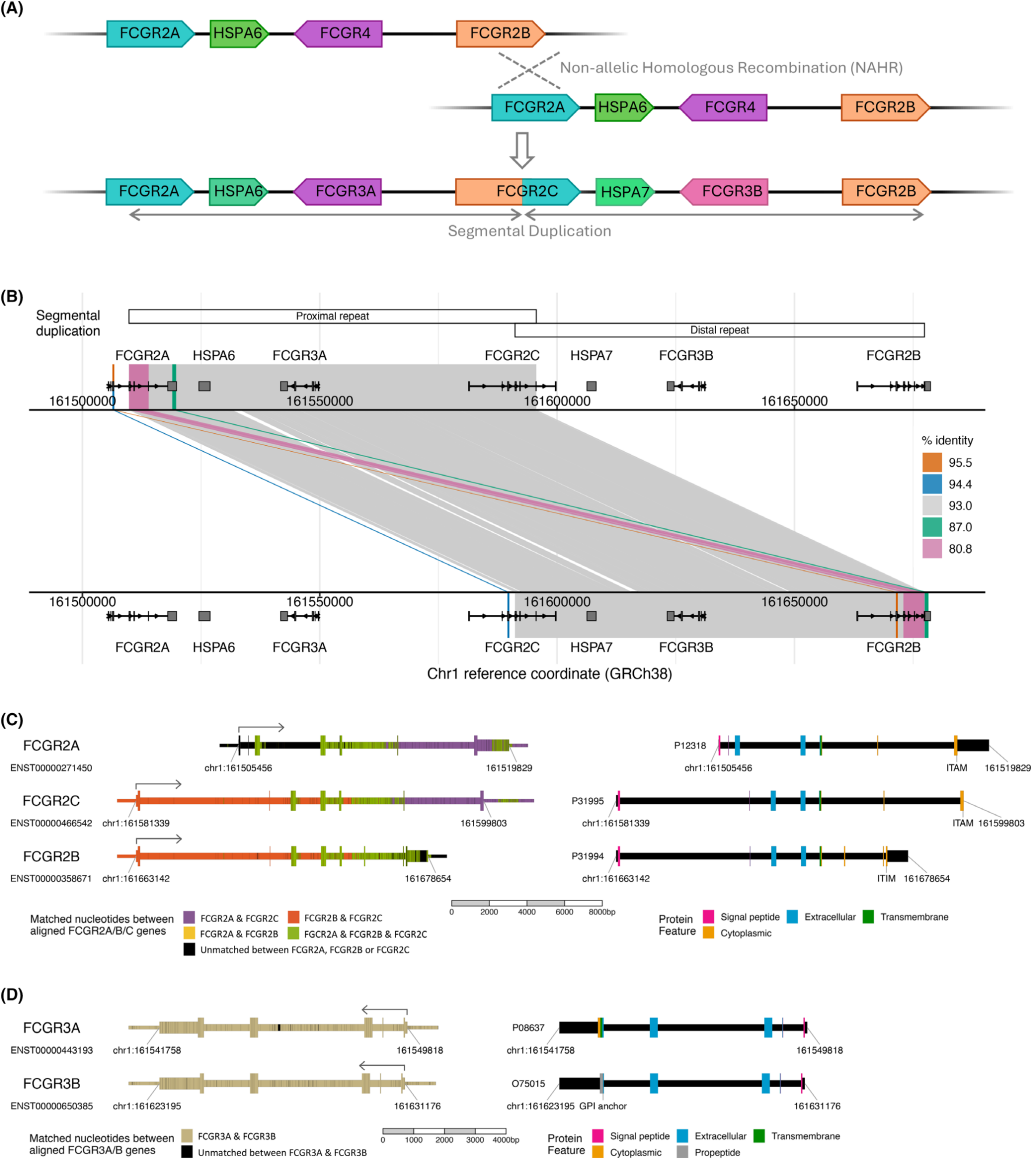
Evolution and homology of the low‐affinity *FCGR* locus. (A) Evolution of the low‐affinity *FCGR* locus in humans. A nonallelic homologous recombination (NAHR) event between *FCGR2A* and *FCGR2B* resulted in the duplication of *FCGR4*. The unequal crossover created the large segmental duplication that is known today and gave rise to the *FCGR3A* and *FCGR3B* genes as well as the emergence of the fusion gene *FCGR2C*. The NAHR event occurred approximately 9 million years ago and humans share the segmental duplication with chimpanzees and gorillas, but not orangutans or macaques. (B) Alignment of the low‐affinity *FCGR* locus to itself demonstrates homology derived from the NAHR event. The human reference sequence (chr1:161,500,000–161,700,000, GRCh38 p.12) was aligned to itself using minimap2 (‐P ‐k19 ‐w19 ‐m50 ‐‐cs = short),^36^ and alignments between different regions of the locus are indicated as diagonal segments. The alignment between identical coordinates is not included and neither are alignments between short interspersed nuclear elements (SINE) in two locations within 4k bp of the start of *FCGR2A* and in the intergenic region between *FCGR3A* and *FCGR2C*. Distinct alignments are indicated by color, insertion/deletions are indicated as diagonal gaps, and percentage identities are given for each alignment. The largest alignment (gray) indicates the segmental duplication regions, and the smaller alignments indicate further homology between *FCGR2A*, *FCGR2B*, and *FCGR2C*; including exon 3, exons 4–6, and part of the 3′‐UTR. Gene structures and locations are shown for MANE select and canonical transcripts and, in concordance with current annotation, *FCGR2A* and *FCGR2C* have a total of seven exons compared with earlier annotations which show eight. This change is due to updated understanding that the sequence previously annotated as exon 6 in *FCGR2A* and *FCGR2C* is spliced out. This exon numbering is continued throughout the figures and review. (C) Gene homology and protein features for *FCGR2A*, *FCGR2B* and *FCGR2C*. Multiple sequence alignments highlight the extensive homology between *FCGR2A*, *FCGR2B* and *FCGR2C*. Gene region reference sequences (GRCh38 p.12) were extended to include flanking regions and aligned to each other using the anchoring‐based CHAOS‐DIALIGN aligner.^37^ An anchor‐based method is required for alignment of short, high‐homology regions (such as exon 3) within longer sequences of lower homology. Matching nucleotides between aligned genes are indicated by color, clearly showing that almost all of *FCGR2C* is homologous to either *FCGR2A* or *FCGR2B*, or both. Additionally, *FCGR2B* has a limited region toward the 3′ end that is not homologous to either *FCGR2C* or both *FCGR2A* and *FCGR2C*. Gene and transcript structure coordinates are for MANE select transcripts (Ensembl IDs indicated) and do not include an annotated 3′‐UTR region for *FCGR2C*. The flanking regions are 1000 bp in both the 5′ and 3′ UTR directions, except for the 3′ end of *FCGR2C* which was extended by 2675 bp to include the region of homology between *FCGR2A* and *FCGR2C* 3′‐UTR. Locations of protein features within gene regions are shown for Uniprot IDs P12318 (FcγRIIa), P31994 (FcγRIIb), and P31995 (FcγRIIc) and genome coordinates are accessible via UCSC Genome viewer data tables. ITAM and ITIM signaling motif coordinates can be determined from the location of consensus sequence motifs.^38^, ^39^, ^40^, ^41^ (D) Gene homology and protein features for *FCGR3A* and *FCGR3B*. Pairwise sequence alignment of *FCGR3A* and *FCGR3B* (GRCh38 p.12) shows the extent of homology between these genes. Gene region sequences were extended to include 1000 bp 3′ and 5′ flanking regions and aligned using ggsearch2seq.^42^ Matching nucleotides in the aligned sequences are shown in beige and these extended gene regions have 96.8% identity. Protein features on genomic coordinates are indicated for Uniprot IDs P08637 (FcγRIIIa) and O75015 (FcγRIIIb) and accessible via UCSC Genome viewer data tables apart from annotation for extracellular and propeptide features, which can be inferred from signal peptide and GPI anchor coordinates.

The human low‐affinity *FCGR* genes are clustered in a single locus on chromosome 1q23.3. This cluster is highly polymorphic and contains homologous sequences with high sequence identity. The genes encoding FcγRIIa (*FCGR2A*) and FcγRIIb (*FCGR2B*) originated from a duplication of an ancestral FcγRII gene before the divergence of primates.^21^ A subsequent segmental duplication occurred in the human‐gorilla ancestor approximately 9 million years ago^21^, ^34^, ^35^ due to nonallelic homologous recombination (NAHR) between *FCGR2A* and *FCGR2B* (Figure 2A). This event gave rise to the FCGR3 genes (*FCGR3A*, *FCGR3B*) and *FCGR2C*, a new hybrid member of the *FCGR2* family, originating from the 5′ end of *FCGR2B* and the 3′ end of *FCGR2A*. Therefore, the chimeric *FCGR2C* gene contains the sequence for an extracellular domain almost identical to that of FcγRIIb but with intracellular ITAM‐containing components homologous to that of FcγRIIa. The homology between these genes is indicated in further detail in Figure 2.

This *FCGR* segmental duplication was also shown to be present in the closest relatives to humans: gorillas and chimpanzees.^34^, ^35^ However, it is absent in orangutans, the remaining members of the Hominidae family, and more distant primates such as gibbons, macaques, and baboons.^26^, ^35^, ^43^, ^44^ There have been conflicting observations regarding the evolutionary timing of this duplication, such as the fact that chimpanzees lack the *FCGR2C* and *FCGR3B* genes^26^ and *FCGR3* expression is detected on sooty mangabey neutrophils.^44^ These discrepancies may have arisen due to copy number variation between different individuals of the same species, or incomplete genome assemblies. The evolution of these genes involved several significant changes, including the insertion of a retroviral element introducing an intracellular exon with a non‐canonical ITAM to *FCGR2A*,^35^ and alterations to the transmembrane^45^ and promoter^46^ sequences of *FCGR3B*, distinguishing its pattern of expression from that of *FCGR3A*.

Identifying the specific immune challenges that shaped *FCGR* evolution is challenging, but studies on mammalian *FCGR3* orthologs indicate that helminth infections exerted significant pressure on the low‐affinity *FCGR* gene family^34^ supporting the hypothesis that balancing immunological responses to varying parasitic burdens has been a critical selective pressure at the *FCGR* loci over evolutionary time. The maintenance of the segmental duplication, especially the emergence and retention of *FCGR3B*, underscores its evolutionary advantage to the species. In contrast, the chimeric *FCGR2C* lacks a definitive functional role and appears to have accumulated mutations that hinder its expression and functionality, such as the exon 3 stop codon, splicing variants, and a compromised 3′‐UTR compared with the *FCGR2A* paralogous sequence.^47^, ^48^ These alterations, which are discussed later, suggest that the *FCGR2C* gene produced by the segmental duplication may be evolving into a non‐functional pseudogene with the classical *FCGR2C*‐ORF haplotype representing the last functional vestige of the original gene.

## 3 *FCGR* GENETICS IN MICE

Understanding the similarities and differences between human *FCGR* genes and those in mice is crucial due to the widespread use of murine models in functional and preclinical studies. Mice differ from humans in both the number of genes and the structure and function of the encoded receptors, including their binding affinities for immunoglobulin.

Initially, it was thought that mice had the same three classes of FcγR as humans—FcγRI, II, and III—each encoded by a single gene.^49^ In contrast, humans have these receptors encoded by eight genes as described above, suggesting that mice did not undergo the same duplication events as humans.^50^ In 2005, a fourth receptor, FcγRIV, was discovered in rodents and some nonhuman primates,^51^ showing high sequence homology to human FcγRIIIa.^52^ In mice, the *Fcgr* genes are located on chromosome 1 (*Fcgr2*, *Fcgr3*, and *Fcgr4*) and chromosome 3 (*Fcgr1*),^50^, ^53^ corresponding to human 1q23 and 1q21, respectively.

Mice, like humans, have only one inhibitory FcγR, FcγRII, which is orthologous to human FcγRIIb and exhibits low‐affinity binding to murine (m) IgG1, IgG2a, and IgG2b,^54^, ^55^ as well as weak binding to mIgE.^56^ mFcγRIII and mFcγRIV also display low‐affinity binding to mIgE,^57^ while none of the human (h) FcγRs have shown affinity for hIgE,^6^ indicating a clear interspecies difference. Humans possess only one high‐affinity FcγR, hFcγRI, whereas mice have two high‐affinity activating receptors: the orthologous mFcγRI,^58^ which binds to monomeric mIgG2a,^6^, ^57^ and mFcγRIV, which also binds monomeric mIgG2a, albeit at a lower affinity than mFcγRI, as well as mIgG2b. Additionally, FcγRI in mice shows low‐affinity binding to IgG2b and IgG3.^54^ The other activating receptor in mice, mFcγRIII, orthologous to hFcγRIIa,^35^ demonstrates low‐affinity binding to mIgG1, mIgG2a, and mIgG2b,^55^ making it unique among murine FcγRs in its ability to bind mIgG1.^54^, ^57^ Cross‐reactivity between the IgG of each species with the other's FcγRs has been well documented.^58^, ^59^, ^60^

The cell‐specific expression patterns of FcγRs differ between the two species. While both humans and mice express hFcγRIIb/mFcγRII in B cells,^22^, ^61^ overall expression in mice is generally broader, with higher levels of the inhibitory receptor expressed on monocytes, macrophages, and neutrophils. In mice, FcγRI expression is restricted to monocyte‐derived dendritic cells (DCs),^62^ a pattern not replicated in humans.^7^ Conversely, the absence of FcγRIIa expression on human natural killer (NK) cells^23^ is not mirrored by the murine ortholog, mFcγRIII.^63^

It is important to note that, although there is reasonably high sequence homology between human and mouse FcγRs,^60^ the defined interspecies orthologs only share around 65% identity.^58^ This disparity is evident in the differences in affinities for immunoglobulins^54^ and expression patterns. Significant variation in IgG affinities for the various FcγRs between mice and humans presents a challenge for determining the efficacy of human tumor‐targeting antibodies in mice.^64^, ^65^ Additionally, there is no widely recognized ortholog of mFcγRIV in humans,^57^, ^58^ nor human FcγRIIa, FcγRIIc, or FcγRIIIb in mice, further limiting the applicability of murine models in inferring the precise roles of human FcγRs and the net result of their engagement in disease. An additional complexity in translating observations into the clinic relates to the extensive *FCGR* sequence variation that exists in humans, both at the level of single‐nucleotide polymorphisms (SNPs) and copy number variation (CNV).

## 4 SEQUENCE VARIATION AT THE *FCGR* LOCUS

### 4.1 FCGR1

Although several *FCGR1A* genetic variants have been identified, they often lack further investigation, thus functionally relevant *FCGR1* variation is not yet well characterized. A SNP was discovered in 1995 within the extracellular domain of FcγRI that replaced an arginine in exon 3 of *FCGR1A* with a termination codon (p.R92X, rs74315310).^29^, ^66^ This mutation has been associated with undetectable expression of FcγRI on phagocytes, without apparent health defects, suggesting possible physiological redundancy. However, this variant was investigated in only one family, limiting broader conclusions.

The impact of three additional *FCGR1A* SNPs on FcγRI effector functions has been explored in vitro.^67^ The nonsynonymous variants p.I301M (rs12078005) and I338T (rs142350980), located in exon 6 and within the transmembrane and intracellular domains of the receptor, respectively,^68^ were found to reduce FcγRI signaling.^67^ The p.V39I (rs7531523) variant, encoding a mutation in the extracellular domain, impaired the receptor's ability to bind IgG in immune complexes. However, since p.V39I is not involved in the IgG‐binding site,^68^ the receptor's ability to bind monomeric IgG remained unaffected.^67^ Although the clinical relevance of these variants has not been investigated and they have low prevalence in the healthy population,^67^, ^69^ as FcγRI has been shown to play an important regulatory role in response to various bacterial and viral infections,^70^, ^71^, ^72^ it could be postulated that *FCGR1A* variants that reduce FcγRI effector functions may be associated with a poorer immune response to infection.

One study utilized a structural prediction model to examine the effect of the four aforementioned *FCGR1A* SNPs (rs74315310,^66^ rs7531523, rs12078005, and rs142350980^67^) in addition to a nonsense variant in exon 5, p.Q224X (rs1338887), which encodes a stop codon in place of glutamine in the third extracellular domain.^68^ While a clear impact of these variants on FcγRI receptor function was not observed, limitations regarding the accuracy of modeling the transmembrane and intracellular domains were reported.

A recent study by Wu et al. identified three novel human *FCGR1A* variants with long‐range PCR and Sanger sequencing.^73^ One SNP in the promotor region, c.‐131C > G (rs1848781), encodes glycine in place of cysteine and significantly increases promoter activity and FcγRI expression on monocytes.^73^ An increase in FcγRI expression has previously been associated with systemic lupus erythematosus (SLE)^74^ and rheumatoid arthritis (RA) progression.^75^ Another SNP, p.D324N (rs1050204), located in exon 6 in the region encoding the FcγRI cytoplasmic domain, was found to enhance phagocytic activity and the production of pro‐inflammatory cytokines in vitro.^73^ Additionally, the *FCGR1A* gene was found to harbor an indel variant, c.845‐23_845‐17delTCTTTG (rs587598788), which involves a 6‐bp sequence in intron 5. This insertion or deletion occurs near exon 6's splice acceptor site, with the insertion variant appearing to correlate with higher levels of FcγRI expression on monocytes. All three variants were also evaluated for associations with sarcoidosis, a disease characterized by abnormal granuloma development.^76^ Wu et al. found a strong association between a specific FCGR1A haplotype (rs1848781C‐rs587598788Del‐rs1050204N) and protection against sarcoidosis development. Furthermore, the c.‐131G and p.324N variants were linked to poorer lung function in sarcoidosis patients.

Evidence indicates that CNV also exists in *FCGR1* genomic regions,^77^ with approximately 2% and 18% of the population possessing deletions or duplications, respectively. However, the precise CNV boundaries are not well‐defined.^32^ Preliminary studies suggest that dosage effects of *FCGR1A* are observed in various cancers, with increased expression associated with better outcomes.^78^ Hence, further research into *FCGR1A* CNVs is required to ascertain their effects and clinical relevance.

Despite being considered a pseudogene^31^ an alternatively spliced variant of human *FCGR1B* lacking exon 5 has been detected in humans.^79^ This variant appears to express a functional receptor capable of binding IgG aggregates in mice, suggesting that the *FCGR1* pseudogenes may have previously unrecognized functions.

### 4.2 Low‐affinity *FCGRs*

**FIGURE 3:**
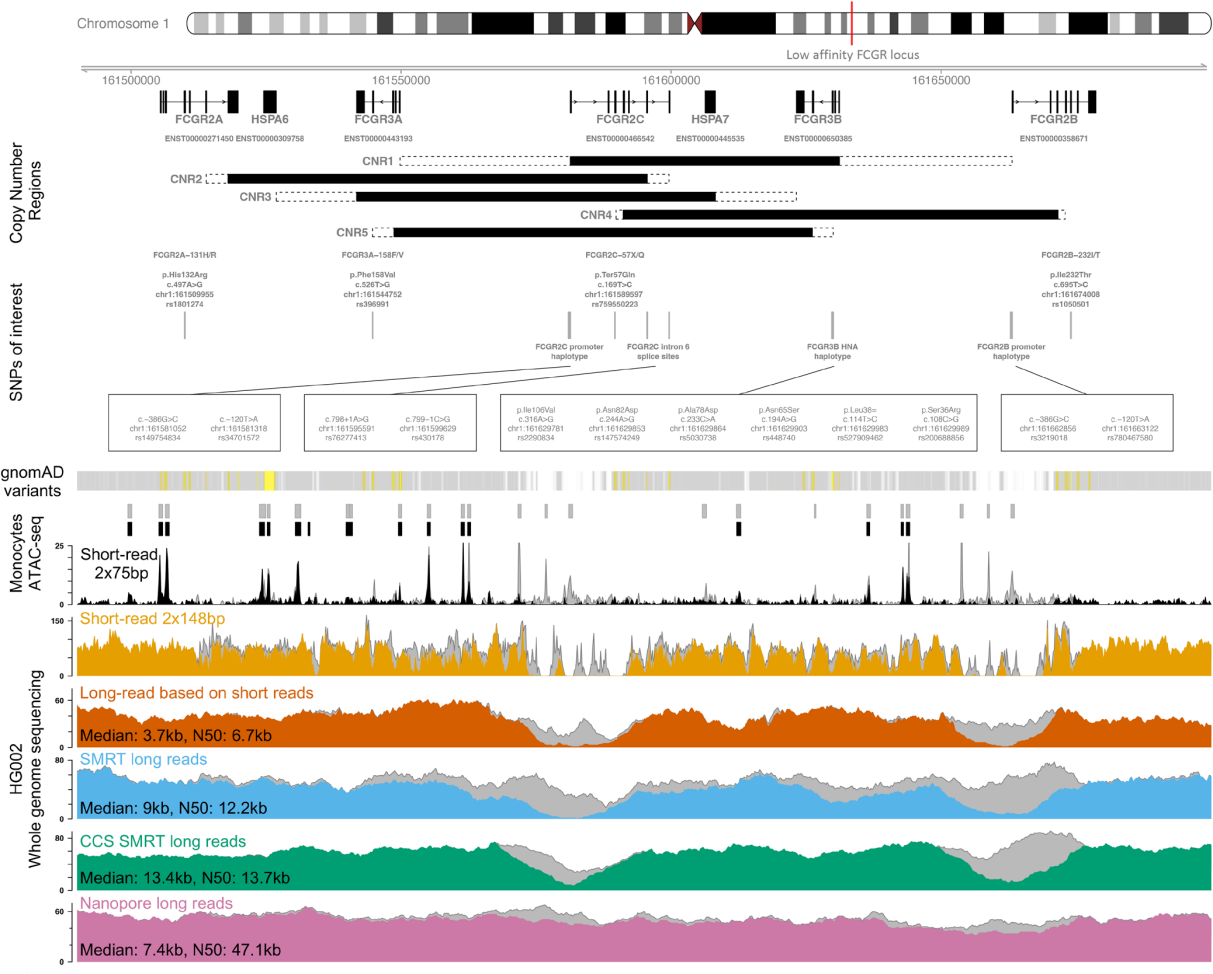
Structure, features, and resolution of the low‐affinity *FCGR* locus. The figure shows the chromosomic position, gene transcripts, CNRs, and SNPs of interest alongside sequencing coverage for the low‐affinity *FCGR* locus using various technologies. The location of the *FCGR* low‐affinity locus is shown within the region chr1:161,490,000–161,700,000 as a red vertical line. Transcript structure and locations are shown for MANE select transcripts, or canonical transcripts where there is no MANE select. Copy number regions (CNR1‐5) are shown with black bars indicating high‐confidence regions of duplication/deletion and dotted outlines indicating regions that may be included in the duplication/deletion. Positions and details of well‐characterized SNPs across the region are indicated as “SNPs of interest” and the track below indicates small variants reported in the gnomAD database. The subsequent tracks are all based on sequencing information from a variety of technologies spanning commonly used read lengths. Technology‐dependent loss of signal occurs in regions of high homology, particularly around *FCGR2C* and *FCGR2B*, where high‐confidence alignments are lacking for the majority of technological approaches. The bottom five tracks display alignment coverage data of the HG002 benchmarking genome from public data resources, utilizing sequencing alignment files provided. Median and N50 read lengths are shown for long‐read technology datasets and calculated using alignments from the locus only. Small variants from the Genome Aggregation Database: There are 47,801 SNVs and indels recorded across the region based on short‐read sequencing‐by‐synthesis of whole genomes and exomes (gnomAD version 4.1.0).^128^ Predicted loss‐of‐function, missense, and synonymous variants are shown in yellow and all other variants are indicated in semi‐transparent gray. Overlaps of multiple variants result in darker gray regions. It should be noted that some variants are potentially missed/misassigned due to homology issues. For example, the well‐characterized *FCGR2C*‐57X/Q variant is not recorded. (Black) Short‐read ATAC‐seq data using sequencing‐by‐synthesis: Data shown is for primary monocytes (Gene Expression Omnibus GSE165997, GSM5059898^129^) and the single sample coverage track indicates open chromatin regions when either all alignments are included (gray, HISAT2 aligner) or only uniquely aligned alignments (black, mapq = 60). Similarly, consensus peaks for the whole dataset are indicated when all alignments are used (gray) or uniquely aligned only (black). Sequencing was performed using 2 × 75‐bp reads on the Illumina NovaSeq platform. (Light orange) Short‐read, sequencing‐by‐synthesis. Genome‐in‐a‐bottle (GIAB) deposited data from the National Institute of Standards and Technology (NIST) utilizing 2 × 148‐bp reads on the Illumina HiSeq platform.^130^ The gray coverage track includes all alignments and orange track indicates the coverage of high‐confidence alignments (mapq = 70). Both coverage tracks are downsampled to 25% of the total data. These alignments utilized the Novoalign aligner and all reported alignments are primary. (Dark orange) Long reads based on short‐read sequencing. Demonstration data are available on application from Illumina, project ID 385613228. Coverage from all available alignments is shown in gray (minimap2 aligner, secondary alignments reported), and from unique alignments only in dark orange (mapq = 60). (Blue) Single‐molecule real‐time (SMRT) long reads. GIAB deposited data from NIST/MtSinai^130^ utilizing PacBio P6‐C4 chemistry and the RS II platform. Coverage when including all alignments is shown in gray (minimap2 aligner, secondary alignments reported), and unique alignments only in blue (mapq = 60). (Green) Circular consensus sequencing (CCS) of single‐molecule real‐time long reads. GIAB deposited data utilizing the PacBio 15‐kb and 20‐kb insert size HiFi reads and Sequel II platform.^131^ Coverage when including all alignments is shown in gray (minimap2 aligner, only primary alignments reported), and unique alignments only in green (mapq60). (Pink) Nanopore long reads. GIAB deposited data by the University of California Santa Cruz (UCSC) utilizing Oxford Nanopore ultra‐long‐read and PromethION platform.^132^ Coverage when including all alignments is shown in gray (minimap2 aligner, secondary alignments reported), and unique alignments only in pink.

Several SNPs have been identified in each of the low‐affinity *FCGR* genes that have functional and/or clinical significance (Table 1 and Figure 3), including effects on auto‐inflammatory and infectious disease susceptibility, and cancer immunotherapy efficacy. In addition, a spectrum of other SNPs exists with unknown impacts on gene expression, protein function, and disease prevalence. NB: Below, we include the reference to the rs number as this is a consistent, searchable descriptor of a SNP independent of isoform and genome assembly.

**TABLE 1:** Overview of genetic variation at the low‐affinity *FCGR* locus.

	Variant		Nucleotide^a^	Amino acid^b^	Impact
	Name	Rs ID			
*FCGR2A*	131H/R	rs1801274	c.497A>G	p.His131Arg	H: Higher affinity for IgG,^6^
					Kawasaki disease,^80^, ^81^, ^82^ childhood ITP,^83^, ^84^, ^85^ Guillain Barre Syndrome,^86^, ^87^ Graves disease,^88^ ulcerative colitis^89^, ^90^, ^91^
					R: Lower affinity for IgG, SLE,^92^, ^93^ sepsis,^94^, ^95^ COVID‐19 death^96^
	62Q/W	rs201218628			
		rs9427397	c.184C>T	p.Gln62Trp	Tryptophan reduces Ca^2+^ signaling^97^, W: KD^98^
		rs9427398	c.185A>G		
Intergenic	2A/3A intergenic (1)	rs2099684	g.161530340A>G	–	Takayasu arteritis^99^, ^100^
	2A/3A intergenic (2)	rs10919543	g.161538827A>G	–	
*FCGR3A*	158F/V	rs396991	c.526T>G	p.Phe158Val	F: SLE susceptibility^93^
					V: Higher affinity for IgG, Ulcerative colitis,^101^ ITP susceptibility,^83^, ^85^, ^102^ Rheumatoid arthritis^103^
	CNV	–	–	–	FcyRIIIa expression, ADCC
					SLE^104^, ^105^
	3A intron 1 enhancer haplotype	rs4656317	g.161549329C>G	–	Alt alleles: Increased FcyRIIIa expression, ADCC^106^, ^107^, ^108^
		rs12071048	g.161549118G>A	–	
	Intragenic haplotype				
	5′‐UTR	rs56199187	g.161551141G>A	–	
	Intron 3	rs77825069	g. 161547895G>T	–	Alt alleles: Enhanced affinity to IgG1, IgG3 and IgG4, Susceptibility to herpes, Epstein–Barr and Varicella zoster^109^
	48 L/R/H	rs10127939	c. 197T>[G/A]	p.Leu48Arg/His]	
*FCGR2C*	Promoter haplotypes	rs149754834	−386G>C	**–**	2B.2 associated with 2C‐ORF
		rs34701572	−120T>A	**–**	
	57X/Q	rs759550223	c.169T>C	p.Ter57Gln	Ref allele: Protein truncated
					Q: Expression of FcyRIIc
					Immune thrombocytopenia,^102^, ^110^ Kawasaki disease,^98^ IgG deficiency^111^
	Intron 6 splice sites	rs76277413	Donor c.798+1	A>G	Only classical FCGR2C‐ORF haplotype associated with FcyRIIc expression^47^
		rs430178	c.799‐1 Acceptor	C>G	
	Thai hap				Variant haplotype: HIV‐1 vaccine efficacy^112^
	Intron 2	rs114945036	126 in In2	C>T	
	Exon 3	rs138747765	c.353	C>T	
	Intron 3	rs78603008	c.391+111	G>A	
	CNV	**–**	**–**	**–**	FcyRIIc expression (ORF only)
					Kawasaki disease^113^
*FCGR3B*	HNA haplotype^c^	rs200688856	c.108C>G	p.Ser36Arg	NA2 associated with SLE susceptibility^93^
		rs527909462	c.114T>C	p.Leu38Leu	
		rs448740	c.194A>G	p.Asn65Ser	
		rs5030738^d^	c.233C>A	p.Ala78Asp	
		rs147574249	c.244A>G	p.Asn82Asp	
		rs2290834	c.316A>G	p.Ile106Val	

	CNV	**–**	**–**	**–**	FcyRIIIb expression, IC uptake, basophil count
Rheumatoid arthritis,^114^, ^115^, ^116^ SLE,^115^ Sclerosis,^117^ Sjogren syndrome,^118^ Ankylosing Spondylitis,^119^ Ulcerative colitis,^101^ Bullous Pemphigoid,^120^ ANCA‐associated vasculitis,^121^ COPD^122^				
*FCGR2B*	2B promoter haplotype	rs3219018	−386G>C	**–**	2B.4 (C and A) increase transcription of *FCGR2B*, 2B.4 associated with SLE susceptibility^123^, ^124^, ^125^
		rs780467580	−120T>A	**–**	
	232I/T	rs1050501	c.695T>C	p.Ile232Thr	T: Excludes FcyRIIb from lipid rafts, SLE susceptibility,^93^, ^126^ malaria protection^127^
	106Del	rs755222686	c.315_318del	pAsn106Del	Del: Increased levels of IgG and abolished binding to IgG1 and IgG3

^a^ Nucleotide numbering reported according to which direction the gene is read in (sense or antisense strand). For example, nucleotides are positive strand from the FCGR2 genes as they are on the forward strand while nucleotides for the FCGR3 genes are on the negative strand as they are on the reverse strand.

^b^ Inconsistencies in the amino acid numbering exist in the literature due to discrepancies in naming based on their position, with some including the signal peptides and others focusing on the mature protein, excluding signal peptides. While the current official HGVS guidelines recommend representing protein‐coding variants in the complete primary translation product, and not a processed, mature, or functional protein, this table follows the amino acid numbering most historically used and adopted by the research community.

^c^ NHA haplotype in the variant name colum for FCGR3B. the footnote explains how the 6 SNPs impact on neutrophil function.

^d^ The SNP that is not involved in distinguishing NA1 from NA2, this SNP is the 1 position that distinguishes NA2 from SH.

### 4.3 FCGR2A

One of the most widely studied SNPs in *FCGR2A* is the A to G transition within exon 4 (rs1801274), resulting in a histidine to arginine substitution (p.H131R) within the IgG‐binding domain.^133^ While both alleles exhibit comparable binding affinities for hIgG3 and hIgG4, the *FCGR2A*‐131H isoform has a higher affinity for hIgG1 and especially hIgG2.^6^ In contrast to the 131R variant, the *FCGR2A*‐131H allele has been shown to facilitate the phagocytosis of IgG2‐opsonized entities,^21^, ^134^ augment neutrophil degranulation, and phagocytosis,^135^, ^136^ and enhance IgG2‐mediated IL‐1β secretion.^137^ Both H and R alleles have been associated with various inflammatory diseases, highlighting the intricate role FcγRIIa plays at the intersect of multiple immunoregulatory pathways. For example, *FCGR2A*‐131H has been associated with increased incidence of Graves' disease,^88^ ulcerative colitis,^89^, ^90^, ^91^ childhood immune thrombocytopenic purpura (ITP),^83^, ^84^, ^85^ and Kawasaki disease (KD),^80^, ^81^, ^82^ whereas *FCGR2A*‐131R has been associated with sepsis^94^, ^95^ and SLE.^92^, ^93^

Additionally, two adjacent SNPs (rs9427397 and rs9427398) within exon three of *FCGR2A* encode a composite SNP (rs201218628) which replaces a glutamine with a tryptophan residue (p.Q62W) in a region of FcγRIIa that is predicted to be a dimerization domain. Initially reported in a common variable immunodeficiency patient cohort, *FCGR2A*‐62W has been connected to reduced calcium signaling and impaired MAPK phosphorylation.^138^ However, its precise functional significance remains elusive as it does not affect *FCGR2A* expression or neutrophil antibody‐dependent cellular cytotoxity (ADCC) capacity.^98^, ^138^ Furthermore, a comprehensive meta‐analysis found a significant association with KD, although its elevated disease risk may result from strong linkage disequilibrium (LD) with other SNPs.^98^

### 4.4 FCGR3A

*FCGR3A* also contains a well‐established SNP within exon 4 (rs396991) that affects the receptor's IgG affinity.^139^ It results in a phenylalanine to valine substitution at amino acid position 158 (F158V) within the second extracellular domain, with the *FCGR3A*‐158V variant exhibiting enhanced affinity for all IgG subclasses.^6^, ^139^ The homozygous *FCGR3A*‐158V/V genotype has been associated with heightened NK cell ADCC capacity,^140^ ITP susceptibility,^83^, ^85^, ^102^ and increased risk of developing RA for individuals of European descent.^103^ However, the lower affinity isoform encoded by *FCGR3A*‐158F has been associated with increased susceptibility for and development of SLE^93^ and lupus nephritis.^141^, ^142^

Within exon 3, there are two alternative alleles that have both been reported to affect FcγRIIIa's affinity for IgG (rs10127939). At amino acid position 48 within the membrane‐distal Ig‐like domain, the reference allele encodes a leucine residue, but substitutions to either G or A can result in an arginine or histidine residue, respectively (*FCGR3A*‐48L/R/H). Both of these variants show increased binding capacity for hIgG1, hIgG3, and hIgG4.^143^ This triallelic SNP has also been associated with the *FCGR3A*‐158V variant whereby the presence of an R or H and at least one copy of the 158‐V allele results in enhanced hIgG‐binding abilities.^144^, ^145^

The *FCGR3A*‐48 L/R/H variant was additionally found to be in LD with two other SNPs and an indel, forming an intragenic haplotype (IH) associated with high FcγRIIIa expression on monocytes and NK cells.^106^ The original *FCGR3A*‐IH described consisted of a 5′‐UTR SNP‐1405A (rs56199187), an intronic indel +690–691InsC (rs33959719), the triallelic nonsynonymous polymorphism 48‐L/R/H (rs10127939), and a second intronic variant +1842T (rs77825069). However, the entry for the intronic indel no longer exists in public databases, suggesting its previous description was a technical artifact.

Furthermore, a study performed on 101 healthy Japanese individuals demonstrated how two SNPs (rs4656317 and rs12071048) located within a potential enhancer region in *FCGR3A* intron 1 were in strong LD with the *FCGR3A*‐158V SNP, and significantly impacted NK cell FcγRIIIa expression and ADCC activity.^107^ It has been suggested this enhancer haplotype (EH) may be responsible for the contrasting findings^146^, ^147^ regarding whether the *FCGR3A*‐158V variant is accountable for changes in expression.^107^

There have also been two intergenic SNPs identified (rs2099684 and rs10919543) between *FCGR2A* and *FCGR3A* that have been associated with the autoimmune disease Takayasu arteritis in Turkish, North American and Chinese Han populations.^99^, ^100^ These variants, which are approximately 11.5 and 3.0‐kb upstream of *FCGR3A*, have been shown to be in high linkage disequilibrium with each other and associated with increased *FCGR2A* expression levels in lymphoblastoid cells.^148^

### 4.5 FCGR2C

As previously mentioned, *FCGR2C* is a fusion gene that is comprised from exons homologous to those from *FCGR2A* and *FCGR2B* (Figure 2C). Despite some in vitro evidence showing FcγRIIc can bind IgG, induce calcium flux, and activate cytotoxic NK cell responses, establishing its true in vivo functional capacity has proven challenging.^47^, ^149^, ^150^ This is partly because it was relatively understudied being previously considered as a pseudogene.^21^, ^151^

A significant proportion (>80%) of the human population harbor a C to T substitution within exon 3, resulting in a stop codon (Ter) at position 57 instead of a glutamine within the open reading frame (ORF).^152^, ^153^ FcγRIIc was first identified on NK cells of individuals with the ORF variant (p.57Gln, *FCGR2C*‐ORF) instead of the stop codon (p.57Ter, *FCGR2C*‐Stop).^149^ However, as most individuals are homozygous for the *FCGR2C*‐57X genotype,^98^ which encodes the truncated isoform, the termination codon is integrated within the current GRCh38 reference genome sequence with the protein‐coding variant described as a SNP (rs759550223).^102^, ^154^ The *FCGR2C*‐ORF variant has been linked to increased susceptibility to several autoimmune diseases, including KD,^98^ ITP,^102^, ^110^ SLE,^155^ and systemic sclerosis,^156^ in addition to IgG subclass deficiency.^111^ It has been reported that the *FCGR2C*‐ORF variant also dictates the expression of FcγRIIc on B cells, reducing the inhibitory effects of FcγRIIb and modulating the overall level of B‐cell activation.^155^ However subsequent, independent research has failed to reproduce the finding of *FCGR2C* expression on B cells.^157^

However, only genotyping *FCGR2C*‐57X/Q is not sufficient to infer FcγRIIc expression as there have been *FCGR2C*‐57Q carriers identified with undetectable levels of cell‐surface FcγRIIc. Instead, they expressed various noncoding *FCGR2C* transcripts that predominantly lacked exon 6 and included a 62‐bp intron 6 insertion.^47^ These aberrantly spliced isoforms were originally attributed to polymorphic donor and acceptor splice sites within intron 6 (rs76277413 and rs430178) that, together with the *FCGR2C*‐57Q SNP, were termed nonclassical *FCGR2C*‐ORF haplotypes (Table 2). However, a more recent study by the same authors only included the non‐canonical donor site to characterize the nonclassical *FCGR2C*‐ORF haplotype.^98^ The non‐functional splice site sequences are incorporated within the GRCh38 reference while the canonical splice sites are annotated as SNPs.

**TABLE 2:** *FCGR2C* haplotypes that impact expression of FcyRIIc.

	*FCGR2C* exon 3 (p.57)	Intron 6 donor splice site (c.798+1)	Consequence
Classical FCGR2C‐ORF	Q	G	FcγRIIc expression
Nonclassical FCGR2C‐ORF	Q	A	No/marginal FcγRIIc expression
FCGR2C‐Stop	X	A or G	No FcγRIIc expression

*Note*: Haplotypes for *FCGR2C* detailing amino acid and nucleotide changes which have consequences for FcyRIIc expression.^47^, ^98^ X represents the termination (Stop) codon. Traditionally only the SNP in exon 3 of *FCGR2C* was genotyped to determine the presence of an open reading frame (ORF); however, for a more accurate prediction of FcγRIIc expression, it is necessary to also genotype the splice sites in intron 6.

Recently, additional *FCGR2C* single nucleotide variants (SNVs) have garnered attention for their potential functional effects, although their definitive biological impact has not yet been conclusively determined. Of particular interest is a haplotype comprising three *FCGR2C* SNVs—c.134‐96C>T within intron 2 (rs114945036), c.353C>T within exon 3 (rs138747765), and c.391+111G>A within intron 3 (rs78603008)—which have been reported to be in complete LD and significantly enhance vaccine efficacy in a cohort of Thai individuals involved in an HIV‐1 vaccine trial.^112^ This haplotype has attracted considerable attention due to its investigation in the only HIV‐1 vaccine trial demonstrating any degree of protection.^158^ However, the functional significance of these variants warrants cautious interpretation as the primers were not *FCGR2C*‐specific,^159^ and a connection with FcγRIIc surface expression could not be elucidated as only one participant in the study harbored the *FCGR2C*‐57Q SNP.

Subsequent investigation of this haplotype within the Black South African population revealed that only the intron 2 SNV (rs114945036) was polymorphic^160^ and, in contrast to the above, was associated with an increased likelihood of HIV‐1 disease progression.^161^ Once again, the FcγRIIc protein was not considered to be involved in the underlying mechanism as only one individual possessed the classical *FCGR2C*‐ORF haplotype. Instead, the authors presented an in silico analysis which supported previous claims that these variants could result in alternative splicing of *FCGR2C* transcripts as a putative CTCF binding motif within a weak enhancer was disrupted. Supporting the notion that these *FCGR2C* variants regulate gene expression, increased levels of the last exon from *FCGR2A* and/or *FCGR2C* were detected in lymphoblastoid B‐cell lines harboring the Thai haplotype SNVs.^162^

The conflicting outcomes in HIV‐1 studies may be due to LD relationships specific to different genetic backgrounds that are not always determined. For example, the intron 2 SNV (rs114945036) was also found to be in LD with a nonsynonymous exon 4 variant and two intron 5 SNVs in the Thai cohort (rs373013207, rs74341264, and rs201984478),^112^ whereas was linked to the *FCGR2C*‐57Q SNP and two intron 1 variants (rs2169052 and rs111828362) in the South African cohort.^161^

### 4.6 FCGR3B

Within exon 3 of *FCGR3B*, which encodes the membrane‐distal Ig‐like extracellular domain of FcγRIIIb, a cluster of polymorphisms collectively represents the human neutrophil antigen (HNA)‐1 system. The most common variants, HNA‐1a and HNA‐1b, are differentiated by five SNPs that lead to four amino acid substitutions and affect N‐linked glycosylation (Table 3). HNA‐1a and HNA‐1b are encoded by the NA1 and NA2 haplotypes, respectively. These variants alter the receptor's affinity for hIgG isotypes, with HNA‐1a having a higher affinity for hIgG1 and hIgG3, resulting in more effective phagocytosis of opsonized targets.^6^, ^135^, ^165^ In vitro experiments have shown that the FcγRIIIb allotype encoded in the NA1 haplotype had approximately double the affinity to monomeric hIgG3 of the NA2 haplotype.^166^ Another reported allotype is HNA‐1c that is encoded by the SH haplotype. The SH variant is identical to NA2 at the aforementioned five SNPs but differs from NA2 at an additional position. The functional consequences of this SNP, and thus difference between HNA1b and HNA1c allotypes, are not well characterized. Several other HNA variants have been identified, although their functional and clinical significance remains to be confirmed.^163^, ^167^, ^168^

**TABLE 3:** Single‐nucleotide‐proteins (SNPs) involved in the *FCGR3B* haplotypes which determine the allotypic variants of the human neutrophil antigen (HNA)‐1 classification system.

Rs ID	*FCGR3B*‐NA1		*FCGR3B*‐NA2		*FCGR3B*‐SH	
	Nucleotide	Amino acid	Nucleotide	Amino acid	Nucleotide	Amino acid
rs200688856	c.108G	Arginine (R)	c.108C	Serine (S)	c.108C	Serine (S)
rs527909462^a^	c.114C	Leucine (L)	c.114T	Leucine (L)	c.114T	Leucine (L)
rs448740	c.194A	Asparagine (N)	c.194G	Serine (S)	c.194G	Serine (S)
rs5030738^b^	c.233C	Alanine (A)	c.233C	Alanine (A)	c.233A	Aspartic acid (D)
rs147574249	c.244G	Aspartic acid (D)	c.244A	Asparagine (N)	c.244A	Asparagine (N)
rs2290834	c.316G	Valine (V)	c.316A	Isoleucine (I)	c.316A	Isoleucine (I)

*Note*: These are the three major haplotypes but typing is considered incomplete because rare cases have been revealed of different combinations of these SNPs and/or additional variants.^163^, ^164^

^a^ rs527909462 is a synonymous variant.

^b^ rs5030738 represents the SNP that is not involved in distinguishing NA1 from NA2; it is the sole position that distinguishes NA2 from SH.

Susceptibility to various autoimmune disorders has been linked to these HNA antigens. The higher affinity HNA‐1a has been associated with an increased risk of a condition marked by FcγR‐mediated neutrophil activation, known as anti‐neutrophil cytoplasmic antibody systemic vasculitis,^168^ whereas the lower affinity HNA‐1b isoform has been linked to SLE development.^169^, ^170^ Additionally, the HNA system holds clinical significance in pregnancy; rare instances of potentially fatal neonatal immune neutropenia have been observed when fetal neutrophils expressing paternal HNA antigens are targeted by maternal antibodies.^171^, ^172^, ^173^

### 4.7 FCGR2B

Unlike the other two *FCGR2* family members which have seven exons, *FCGR2B* has eight exons that are alternatively spliced. The predominant FcγRIIb isoforms, FcγRIIb1 and FcγRIIb2, exhibit distinct localization patterns and functional properties. FcγRIIb1, transcribed from all eight exons, is the only FcγR routinely expressed in B cells and functions to inhibit B‐cell receptor (BCR) signaling, thus limiting B‐cell expansion and activation in the presence of antigen‐specific ICs.^174^ In contrast, FcγRIIb2 has the 57‐bp exon 6 spliced out from between the transmembrane domain and cytoplasmic ITIM. It is the primary transcript expressed in myeloid cells and is mainly involved in endocytosis of ICs.^38^ Exon 6 of *FCGR2B*, present in FcγRIIb1, enhances B‐cell membrane tethering, whereas its absence from FcγRIIb2 allows for rapid internalization.^175^ This exon does not have a corresponding exon in the current annotation for *FCGR2A* or *FCGR2C* (Figure 2C).

A well‐studied polymorphism within exon 5 of *FCGR2B* (rs1050501), involving a T to C substitution, has attracted attention for its impact on FcγRIIb's inhibitory signaling and its potential to disrupt immune system balance. This substitution, occurring at amino acid position 232 within the transmembrane region and resulting in an isoleucine to threonine change (I232T), was initially identified in a Japanese cohort of SLE patients.^176^ The *FCGR2B*‐232T variant has been shown to interfere with FcγRIIb's ability to attenuate the activating signaling of the BCR by impeding its association with lipid rafts in the plasma membrane.^177^, ^178^ Moreover, as FcγRIIb is also expressed on dendritic cells (DCs), the presence of FcγRIIb‐p.Thr232 can also impact DC maturation and consequently T‐cell stimulation.^179^, ^180^, ^181^ In multiple studies across numerous ethnic backgrounds, homozygosity of the *FCGR2B*‐232T variant has been strongly associated with SLE.^8^, ^93^, ^126^, ^176^, ^182^, ^183^, ^184^, ^185^ Conversely, a T/T genotype was also shown to provide a protective effect against severe malaria infection in an East African population.^127^

Allelic variation can also impact the expression levels of the FcγRs. Notably, two crucial SNPs reside in the promoter regions of both *FCGR2B* and *FCGR2C*, positioned at −386 and −120 relative to the translational start site (Table 4). At the −386 position, these SNPs can either be G or C, while at the −120 position, they can be A or T, resulting in four distinct haplotypes named 2B.1, 2B.2, 2B.3, and 2B.4. The 2B.1 haplotype is prevalent in both *FCGR2C* and *FCGR2B* promoter regions, while the 2B.2 and 2B.4 haplotypes have only been reported upstream of *FCGR2C* and *FCGR2B*, respectively. To date, there has never been reported a case of the 2B.3 haplotype of −386G with −120A. With a luciferase reporter assay, the rarer *FCGR2B*.4 haplotype was demonstrated to increase *FCGR2B* transcription in comparison with the more common *FCGR2B*.1 haplotype.^123^ This observation was then supported when transcription factor (TF) binding and FcγRIIb expression were seen to be enhanced in primary B cells with the *FCGR2B*.4 haplotype.^186^ Augmented transcriptional activity from 2B.4 has also been observed in neutrophils and monocytes.^124^, ^187^ The 2B.4 haplotype has also been associated with increased SLE susceptibility.^123^, ^124^, ^125^

**TABLE 4:** Promoter haplotypes for *FCGR2B* and *FCGR2C.*

Promoter haplotype	c.−386	c.−120	Found upstream of
2B.1	G	T	*FCGR2B* and *FCGR2C*
2B.2	C	T	*FCGR2C*
2B.3	G	A	Not reported
2B.4	C	A	*FCGR2B*

*Note*: Promoter haplotypes for *FCGR2B* and *FCGR2C* detailing the nucleotide changes and genes effected.^123^ Haplotypes comprised of two SNPs at nucleotide positions −386 and −120 relative to the start of translation in homologous promoter regions of *FCGR2B* and *FCGR2C*. Specific haplotypes are associated with particular genes and the 2B.3 haplotype has never been reported.

Additionally, a recently described in‐frame deletion of codon AAT within exon 3 (rs755222686, p.Asn106del) was shown to completely eradicate FcγRIIb binding with hIgG1 and hIgG3.^188^ While this non‐functional protein resulted in heightened levels of hIgG, it has only been reported in a very small subset of the Icelandic population so far.

## 5 STRUCTURAL VARIATION AT THE *FCGR* LOCUS

### 5.1 History

CNV involving *FCGR* genes have been notoriously challenging to characterize due to the genomic complexity of these loci and limitations in available technologies. The first report of CNV at the *FCGR* locus dates back to the 1990s when a homozygous deletion of *FCGR3B* was observed in mothers of children with neonatal immune neutropenia.^171^, ^172^, ^173^ This deletion was subsequently found to be linked with *FCGR2C* CNV.^189^, ^190^ To investigate associations with autoimmune diseases, methods such as array comparative genomic hybridization (aCGH) and restriction enzyme digest variant ratio assays were developed, and large‐scale studies examining global CNVs suggested that *FCGR2A* and *FCGR2B* were also candidate genes for duplication or deletion events.^191^, ^192^, ^193^, ^194^

However, in 2008 the multiplex ligation‐dependent probe amplification (MLPA) assay was developed to study the genetic variation at the *FCGR* locus in a single assay.^102^ It was hypothesized that the variation captured by both CNV and SNPs could collectively encode a “susceptibility phenotype” for autoimmune/inflammatory diseases as the balance between activating and inhibitory signaling was altered by genomic variants. The MLPA assay was conducted on >600 individuals to demonstrate extensive variation at the *FCGR* cluster, but showed only *FCGR3A*, *FCGR2C*, and *FCGR3B* were subject to CNV—and not *FCGR2A* or *FCGR2B* as previously reported.^102^, ^154^

In 2012, a study by Machado et al. utilized Sanger sequencing of PCR amplicons to investigate the NAHR events generating *FCGR* CNV and localized the breakpoints to distinct hotspots.^34^ This analysis involved identifying and utilizing paralogous sequence variants (PSVs), which are single nucleotide differences between the two paralogous repeats of the segmental duplication. However, in 2013, Mueller et al. examined the genetic pathology of *FCGR3B* deletions and reported that 36% of the candidate PSVs were polymorphic, compromising any breakpoint analysis that used them.^159^ Mueller et al. also refined the boundaries of the *FCGR2C/FCGR3B* CNV to two highly paralogous 24.5‐kb blocks covering the 3′ ends of *FCGR2C* and *FCGR2B*, which they stated were devoid of true PSVs not subject to allelic variation. In 2017, Rahbari et al. reanalyzed the same fosmid sequences and were able to further define the breakpoints within this 24.5‐kb block, attributing the discrepancies to the consideration of the duplications present.^114^

### 5.2 Structure

MLPA analysis of >4000 individuals was conducted in 2015 and four distinct CNV regions (called CNRs) were described, mapping the most common areas of gain or loss within the low‐affinity *FCGR* locus.^48^ The PSVs utilized in this MLPA assay included 7 pairs that were designed to distinguish between the two 82‐kb paralogous repeat units, four of which were designed to detect nonpolymorphic PSVs. During this work, the breakpoints of the CNR were then further refined, documenting the generation of functional chimeric genes and the novel fourth variant CNR4 for the first time. This study also provided support for the idea that there is population variation in *FCGR* copy number profiles.^194^ Briefly outlined below (and depicted in Figure 3) is the current understanding of the structure of the CNRs, where the numbering reflects their prevalence.

- CNR1 includes *FCGR2C*, *HSPA7*, and *FCGR3B* with breakpoints thought to occur within the intergenic region between *FCGR3A*‐*FCGR2C* and *FCGR3B*‐*FCGR2B*.
- CNR2 has intragenic breakpoints in intron 6 of *FCGR2A* and *FCGR2C*. It encompasses the entire genomic sequence of the *HSPA6* and *FCGR3A* genes as well as the last exon of *FCGR2A* and all but the last exon of *FCGR2C*.
- CNR3 includes *FCGR3A*, *FCGR2C*, and *HSPA7* with the breakpoints within the intergenic region between *HSPA6*‐*FCGR3A* and *HSPA7*‐*FCGR3B*. Like CNR1 and CNR2, both deletions and duplication of CNR3 have been observed.
- CNR4 has intragenic breakpoints and includes the entire genomic sequence for the *HSPA7* and *FCGR3B* genes. Its start includes exon 4 of *FCGR2C*, and its end contains the proximal part of *FCGR2B* including exon 3. Only deletions of CNR4 have been reported.
- CNR5, a novel CNR, was reported in 2021 in some individuals indigenous to the Ecuadorian highlands.^195^ The authors showed that the CNR deletion spanned from the third intron of *FCGR3A* to the third intron of *FCGR3B*, resulting in the creation of a *FCGR3B*/A chimeric gene which was associated with FcγRIIIa and FcγRIIIb deficiency.

Considering that *FCGR* CNV is facilitated by recurrent NAHR between the highly homologous repeats of the segmental duplication at a high mutation rate,^34^ in addition to the development of increasingly sophisticated technologies which exhibit enhanced fidelity, it is anticipated that more CNRs will be discovered as the *FCGR* locus is explored within larger and more diverse cohorts.

### 5.3 Functional consequences

The CNRs at the *FCGR* locus can have significant functional consequences for FcγR expression, and thus the response to IgG, through two main mechanisms: (i) changes to gene dosage and (ii) the introduction of chimeric genomic sequences.

Gain of CNRs that result in increased *FCGR3B* gene copies leads to an augmentation of *FCGR3B* mRNA levels,^98^ and protein expression at the cell surface.^124^, ^154^, ^189^, ^190^ Increased expression of FcγRIIIb has been shown to enhance the ability of neutrophils to bind and uptake immune complexes.^196^ Recke and co‐workers also reported elevated ROS production in donors with *FCGR3B* CNV.^120^ Loss of the same CNRs, such as CNR1 or CNR4, have been shown to reduce FcγRIIIb expression and function on neutrophils,^170^, ^196^ associated with the impairment of IgG‐mediated opsonization.^171^, ^197^ Similarly, extra copies of *FCGR3A* (through duplication of CNR2 or CNR3) have been linked with increased NK cell‐surface expression of FcγRIIIa; however, the association is not as clear compared with the *FCGR3B* effects.^98^, ^154^ CNV of *FCGR2C* has also been linked to changes in expression, but only in individuals that harbor the *FCGR2C*‐ORF SNP.^98^, ^102^

As previously mentioned, CNRs do not just result in expression changes due to differences in gene dosage but can also create chimeric gene products and cause the rearrangement of key cis‐acting regulatory elements. For example, deletion of *FCGR3B* has been reported to generate a chimeric event which results in aberrant regulation of *FCGR2B* transcription. The deletion causes the *FCGR2C* regulatory sequence to juxtapose the coding sequence of *FCGR2B*, placing *FCGR2B* expression under the control of the 5′ flanking sequence of *FCGR2C*.^159^ This results in ectopic accumulation of FcγRIIb on NK cells that apparently impairs ADCC, potentially providing an explanation for SLE susceptibility.^47^, ^159^

Furthermore, deletion and duplication of CNR2 have both been shown to create chimeric gene products.^184^ The novel chimera generated by CNR2 deletion is comprised of the first seven exons of *FCGR2A* followed by exon 8 and the 3′‐UTR of *FCGR2C*.^48^ Interestingly, despite the distal part of *FCGR2C* being directly derived from *FCGR2A* during the segmental duplication event, the *FCGR2A*/2C chimeric gene product was functionally distinct from the wild type FcγRIIa. It is expressed at significantly lower levels on neutrophils and monocytes thus reducing the ability to induce a response to IgG.^48^ In contrast, CNR2 duplication can create the inverse *FCGR2C*/2A chimeric product that consists of the first seven exons of *FCGR2C* followed by exon 8 and the 3′‐UTR of *FCGR2A*. This can result in increased FcγRIIc expression at the cell surface but only if the *FCGR2C*‐ORF SNP coexists on the same haplotype. The difference in expression was attributed to the 3′‐UTR of *FCGR2A* providing increased stability to the mRNA molecule compared with the 3′‐UTR of *FCGR2C*.

The CNR4 deletion can result in an *FCGR2B* null variant, resulting in loss of the first three exons of *FCGR2B* to be replaced with exons 1–3 of *FCGR2C*—potentially introducing the *FCGR2C*‐stop codon in exon 3. When this polymorphic stop codon from *FCGR2C* is used, it effectively silences the inhibitory FcγRIIb gene and causes expression of FcγRIIb to be reduced by half in heterozygous individuals.^48^

### 5.4 Disease consequences

The main rationale for the previous studies was to elucidate the structure of the *FCGR* locus under different copy number states and link variants to cellular responses, pathology, and disease implications. Deletions of *FCGR3B* have previously been associated with the susceptibility to numerous autoimmune diseases: glomerulonephritis,^192^ SLE,^34^, ^115^, ^121^, ^159^, ^170^, ^185^, ^196^, ^198^ ulcerative colitis,^101^ rheumatoid arthritis (RA),^114^, ^116^, ^199^, ^200^, ^201^ ankylosing spondylitis,^119^ systemic sclerosis,^117^ primary Sjögren syndrome,^118^, ^200^ microscopic polyangiitis, and Wegener's granulomatosis.^121^ The more recently described CNR4 deletion is reported to be very rare, detected in only 0.1% (5/4357) of the study population, and does not necessarily contribute to autoimmune disease as 3 of the 5 individuals with the variant were healthy adults. However, the other two cases were patients with vasculitis and SLE, so it was suggested loss of CNR4 may predispose individuals to autoimmunity.^48^ Similarly, knockout mice that lack FcγRIIb are viable but are prone to severe, early onset autoimmunity,^202^, ^203^ but only in certain genetic contexts that exhibit higher disease incidence.^204^

The data available on the association of *FCGR3A* CNV and autoimmune diseases have discrepancies, with loss of *FCGR3A* being associated with RA and SLE in Taiwanese cohorts^104^ but not in individuals of European descent.^199^ These contradictions could be the result of true differences between populations, small cohort sizes, or limitations of the methodologies. A recent UK Biobank study, that investigated CNVs, which modify protein products, showed a significant association between *FCGR3B* copy number and increased basophil count.^122^ A reduction in *FCGR3B* gene dosage was linked with a higher risk of developing chronic obstructive pulmonary disease (COPD). However, it was also noted that while the haplotype‐informed analysis could differentiate between the *FCGR3A* and *FCGR3B* paralogs, other functional *FCGR* SNPs/CNRs could be the causal variants for other associations (e.g., lymphocyte, monocyte, and eosinophil counts) at the locus.

## 6 LINKAGE DISEQUILIBRIUM AND POPULATION VARIATION

Additional considerations for the interpretation of genetic variants at the low‐affinity *FCGR* locus are (i) the significant variation across different ethnic backgrounds and (ii) the pronounced extent to which LD is present. Due to the variants being in such close physical proximity to each other, they are prone to co‐inheritance and thus certain combinations co‐exist on the same allele more frequently than would be expected by chance.

In 2019, Nagelkerke et al. published a comprehensive overview of *FCGR* variant frequency and LD patterns, based on data from over 1800 healthy individuals from African, Chinese, and European populations.^98^ Significant population differences were found in the frequencies of all genotyped SNPs and CNRs, except for the *FCGR3A*‐158V variant, which was largely similar across groups. Within the European and African populations, further analysis revealed subtle distinctions among Europeans and notable differences between individuals of Antillean, Ethiopian, South African, Surinamese, and West African descent.

A key finding across all populations was the difference in *FCGR2C* haplotype prevalence. For instance, the nonclassical *FCGR2C*‐ORF haplotype was more common in African populations than in Europeans, while the classical *FCGR2C*‐ORF variants were present in 11% of European alleles but were uncommon in those of African descent and almost entirely absent in the Chinese population. When examining the LD landscape in a CNR‐neutral context, the classical *FCGR2C*‐ORF variants were shown to be in LD with numerous other *FCGR* SNPs. In Europeans, they were almost completely in LD with the *FCGR2C* 2B.2 promoter haplotype, in strong LD with the *FCGR2A*‐27W and *FCGR2B* 2B.4 variants, and in weak LD with the higher affinity *FCGR3A*‐158V SNP. Similar, albeit weaker, LD relationships were also identified in the African and Chinese cohorts carrying the classical *FCGR2C*‐ORF haplotype.

These findings^98^ were largely in agreement with previously reported *FCGR* variant frequencies and LD patterns,^34^, ^110^, ^160^, ^184^, ^205^, ^206^ yet expanded the range of variants and ancestry‐specific effects considered. For example, the LD detected between the high‐affinity *FCGR2A*‐131H and *FCGR3A*‐158V alleles were consistent with previous conclusions made in African and European cohorts,^184^, ^205^ yet were actually inverted in the Chinese population with LD between the higher affinity *FCGR2A*‐131H and the lower affinity *FCGR3A*‐158F alleles.

LD was also explored between the SNPs and CNV, revealing that CNR1 was in strong LD with the nonclassical *FCGR2C*‐ORF haplotype in both European and African populations, and with the *FCGR3B*‐SH haplotype in Europeans. *FCGR2A*‐H131R, *FCGR3A*‐V158F, and *FCGR2B*‐I232T also displayed association with CNR1 CN changes. LD was only observed for CNR2 between *FCGR2B*‐I232T in Europeans, and no statistically significant LD was found for CNR3.

While this research provided insight into LD and population variation, the field still lacks an unbiased understanding of the low‐affinity *FCGR* locus as only previously studied and confirmed as functionally relevant variants were genotyped. The MLPA assay, which is widely used and regarded as the most effective high‐throughput method for determining *FCGR* variants,^207^ only examines eight of the previously detailed SNP/haplotypes (along with CNR1‐4) which, although important as associated with autoimmune/infectious diseases, does not offer a complete picture. For instance, the *FCGR3A*‐48 L/R/H polymorphic site described above was not assessed but is a good example of why detailed knowledge of all *FCGR* LD is critical for accurate interpretation. The triallelic *FCGR3A* variant was first recognized in 1996 where it was observed that *FCGR3A*‐48R and *FCGR3A*‐48H variants had higher binding capacity for hIgG compared with the more prevalent wildtype FCGR3A‐48L allele.^208^ However, this difference in hIgG binding was quickly ascribed solely to *FCGR3A*‐158F/V alleles as a consequence of LD.^209^ In 1997 the same authors demonstrated a distinct linkage between the lower affinity *FCGR3A*‐158F and *FCGR3A*‐48L wildtype alleles, and that NK cells with the *FCGR3A*‐158V variant bound significantly more IgG irrespective of the *FCGR3A*‐48L/H/R genotype.^209^ However, this claim was challenged when revisited in 2014 and FcγRIIIa expression was appropriately controlled for.^145^ It was shown that although *FCGR3A*‐158V is strongly linked with both *FCGR3A*‐48H and R variants, the *FCGR3A*‐48L/H/R genotype can independently influence IgG affinity. Specifically, the 158V/48R haplotype exhibited the highest IgG‐binding affinity, and both the 158V/48R and 158V/48H haplotypes displayed significantly greater binding capacity compared with the 158V/48L combination. Given that both the biallelic *FCGR3A*‐158F/V and the triallelic *FCGR3A*‐48L/R/H polymorphic positions can independently influence FcγRIIIa hIgG binding, alongside alterations in expression levels resulting from CNR linkage and the remaining *FCGR3A*‐IH variants, it is imperative that appropriate genetic controls and stratified groups are implemented to incorporate all known factors.

## 7 INFLUENCE OF *FCGR* GENETICS ON COVID‐19

In addition to the multiple associations that the *FCGR* genes have with autoimmune diseases, they also have important roles in regulating responses to infection. Most recently this has been demonstrated in their influence on SARS‐CoV‐2 control and disease outcome,^210^, ^211^, ^212^ whereby failure to coordinate efficient binding to SARS‐CoV‐2‐specific Abs and subsequent effector functions has been correlated with increased mortality.^213^ Considering the growing concerns about SARS‐CoV‐2 variants impacting vaccine efficacy by evading antibody neutralization, there is an expanding interest in maintaining and augmenting protection mediated by FcyR effector functions.^214^, ^215^, ^216^, ^217^ While it has been reported that FcyR effector activities, such as viral uptake into phagocytic cells and increased inflammation, may also heighten the pathophysiology of the virus through antibody‐dependent enhancement of infection (ADE),^218^, ^219^, ^220^ there is currently no evidence from preclinical or clinical studies that SARS‐CoV‐2 vaccines elevate the risk of ADE.^221^, ^222^, ^223^ Furthermore, a single‐cell RNA‐seq analysis of immune cells identified a distinct absence of global induction of the interferon‐stimulated genes (ISGs) program in severe COVID‐19 patients, in contrast to its presence in mild cases, which was attributed to FcyRIIb‐mediated antagonism of interferon receptor signaling.^224^ This altered myeloid transcriptional state, which serves as a COVID‐19 severity hallmark,^225^ implies the clinical blockade of FcyRIIb with antibodies may be a potential means to reverse the inhibition of ISGs and diminish severe responses.^224^

Among factors such as antibody isotype, glycosylation, and vaccine regimen, *FCGR* polymorphisms have also been reported to influence host immunological response to SARS‐CoV‐2. For example, the homozygous *FCGR3A*‐158V/V genotype was shown to be overrepresented in COVID‐19 patients that were hospitalized or subsequently died, whereby NK cells expressing the higher affinity (*FCGR3A*‐158V/V or V/F) demonstrated significantly higher pro‐inflammatory ADCC responses that may have contributed to immunopathogenesis.^226^ However, another study by López‐Martínez et al. evaluated the impact of both *FCGR2A*‐131H/R and *FCGR3A*‐158F/V SNPs on COVID‐19 severity, only found the *FCGR2A*‐131R allele to be significantly associated with an increased risk of mortality.^96^ This polymorphism, encoding the FcγRIIa isotype with lower IgG affinity, had also previously been associated with more severe SARS‐CoV‐1 infection.^227^

## 8 IMPACT OF *FCGR* GENOTYPES ON IMMUNOTHERAPY

It has been proposed that *FCGR* genotyping can be leveraged for precision medicine in predicting response to mAb immunotherapy. The two *FCGR* variants that have gained the most attention in this regard are the *FCGR2A*‐131H/R and *FCGR3A*‐158F/V SNPs which alter FcγR hIgG affinity as detailed above. In the autoimmune disease setting, the *FCGR3A*‐158V variant has been associated with improved rituximab response in ITP,^228^ SLE^229^, ^230^ and RA.^231^, ^232^, ^233^, ^234^, ^235^ Similarly, the *FCGR2A*‐131R variant was associated with favorable responses to adalimumab in RA^236^, ^237^ and with better outcomes for liver transplantation patients treated with rituximab.^238^

Similar associations with efficacy were previously made in the treatment of malignancies. However, as the influence of *FCGR* variants on mAb efficacy has been more extensively investigated, the literature has become less clear and in places directly conflicting. In part, this discordance may reflect both the nature of the studies undertaken (size, real‐world versus clinical trial etc.), as well as the burgeoning number and type of mAb immunotherapy agents and greater diversity in their mechanisms of action.

With respect to direct targeting or cytotoxic antibodies, it is well established that the FcγRs are key determinants of success, whereby FcγR expression profile (A/I ratio), mAb isotype, and FcγR interaction influence therapeutic efficacy.^3^, ^239^, ^240^ Here, the primary effector mechanisms are thought to be ADCC and antibody‐dependent cellular phagocytosis (ADCP).

Initial studies supported that the alleles encoding the high‐affinity alleles *FCGR2A*‐131H and *FCGR3A*‐158V significantly augment cancer patient responses and outcomes to mAb‐based regimens. Firstly, in lymphomas treated with rituximab, both genotypes have independently been associated with higher response rates and longer progression‐free survival (PFS) in follicular lymphoma (FL) (Weng and Levy 2003) and the *FCGR3A*‐158V variant has been associated with favorable clinical response rates in FL,^241^, ^242^ mantle cell lymphoma (MCL),^243^ diffuse large B‐cell lymphoma (DLBCL),^244^, ^245^ and non‐Hodgkin lymphoma (NHL) in general.^246^ These latter findings supported the greater ADCC activity seen for NK cells with the *FCGR3A*‐158V variant, with the mechanism for the *FCGR2A*‐131H being less apparent (for hIgG1 therapeutics for which the affinity is relatively similar to *FCGR2A*‐131R).

Among solid tumors, these findings were extended to trastuzumab in breast^247^, ^248^, ^249^, ^250^ and gastric^251^ cancers, farletuzumab for ovarian cancer,^252^ as well as cetuximab for colorectal cancer^253^, ^254^, ^255^, ^256^, ^257^, ^258^, ^259^ and head and neck squamous cell carcinoma (HNSCC).^260^ Of interest, in renal cell carcinoma patients the high‐affinity genotypes (plus the *FCGR2C*‐57Q variant) were associated with significantly increased tumor shrinkage and overall survival in response to a non‐antibody immune therapeutic, high‐dose aldesleukin (HD‐IL2).^261^

However, several other studies have cast doubt on the prognostic significance of these variants, finding no association between high‐affinity genotypes and clinical outcomes in colorectal cancer,^253^, ^255^, ^262^, ^263^ FL,^264^, ^265^, ^266^ DLBCL,^267^, ^268^, ^269^ and CLL.^270^, ^271^ Moreover, beyond the relatively small‐scale, retrospective studies, the lack of association with clinical impact was also reproduced in studies of clinical trials with much larger cohorts for FL,^272^, ^273^, ^274^ DLBCL,^274^, ^275^ and renal cell carcinoma.^276^

To add to the complexity, there are also a limited number of studies concluding the lower affinity genotypes are associated with better responses to mAb immunotherapy, including within colorectal cancer,^262^, ^277^, ^278^, ^279^ DLBCL,^275^ multiple myeloma,^280^ and breast cancer.^281^

Due to these highly variable findings, the utility of *FCGR* polymorphisms as predictive biomarkers remains inconclusive. The discordance between studies may be attributed to numerous factors, involving differences in disease subset, clonal evolution, treatment regimen, study design, genotyping technique, and sample sizes. Furthermore, a substantial limitation of the aforementioned studies is the majority typically only genotype the two SNPs *FCGR2A*‐131H/R and *FCGR3A*‐158F/V—completely overlooking CNV and other *FCGR* SNPs (which they may be in LD with) that also have the potential for impacting treatment response as detailed above.

Beyond direct targeting mAbs, immunomodulatory mAb therapies—such as agonist antibodies and checkpoint inhibitors—are also impacted by FcγR engagement.^282^, ^283^ In preclinical mouse models, therapeutic efficacy of mAbs directed to immunomodulatory receptors such as GITR, OX40, CD25, and CTLA4 in part are reported to stem from their ability to eliminate intratumoral T regulatory cells (Tregs), thereby unleashing antitumor immunity driven by CD8 T cells.^284^, ^285^, ^286^, ^287^, ^288^, ^289^ In advanced melanoma patients, the *FCGR3A*‐158V variant has been linked to improved responses to the anti‐CTLA4 mAb ipilimumab.^290^ The *FCGR3A*‐158V SNP was found to be associated with significantly improved overall survival in one dataset,^291^ while a meta‐analysis also reported an association with higher response rates,^290^ although it should be noted both studies are small and require further validation.

## 9 ROLE OF *FCGR2B* IN CANCER AND ASSOCIATIONS WITH TREATMENT RESISTANCE

Although mAb immunotherapy has revolutionized cancer treatment,^240^ patients often experience intrinsic or acquired resistance, thus relapse is still a significant obstacle. The mechanisms of action and hence resistance vary with different types of therapeutic antibody and are incompletely resolved, but deeper understanding is central to improved clinical outcomes.^282^ B‐cell malignancies are routinely treated with anti‐CD20 mAbs such as rituximab, which engage FcγR‐mediated effector functions to elicit their therapeutic effects.^292^ One mechanism of resistance to rituximab involves high *FCGR2B* expression.^293^, ^294^, ^295^ Being the predominant IgG receptor expressed on normal and malignant B cells, FcγRIIb facilitates internalization of CD20:rituximab:FcγRIIb complexes through a cis configuration resulting in loss of antibody from the cell surface, heightened mAb consumption, and abrogation of all FcγR‐mediated effector functions.^296^, ^297^ Retrospective analyses found that MCL patients treated with rituximab immunochemotherapy had shorter PFS when their tumor biopsies were FcγRIIb‐positive,^293^ with high FcγRIIb expression on FL also associated with lower response rates with rituximab monotherapy.^294^

Further support for the negative association of FcγRIIb and treatment success arises from genetic studies. Investigations into genes impacted by 1q21‐23 rearrangements, a common aberration in hematological malignancies associated with poor prognosis,^298^, ^299^ identified *FCGR2B* as a gene targeted by the rearrangement leading to deregulated expression.^300^, ^301^ Despite involving different chromosomal partners, the translocations examined in the separate studies resulted in overexpression of the FcγRIIb2 isoform, relative to the expected FcγRIIb1 isoform, on the tumor B cells. The overexpression of FcγRIIb2, the isoform that more readily undergoes internalization, potentially compromises the regulatory function of FcγRIIb, leading to enhanced evasion of antitumor mechanisms and may represent a secondary genetic event that provides additional growth advantage.^301^ FcγRIIb2 was also identified as enriched relative to FcγRIIb1 in CLL cells,^293^ although signaling through the intracellular tail was shown to be redundant for the rituximab internalization process.^302^

A recent study also identified *FCGR2B* as a potential novel oncogene in a germinal center B‐cell (GCB) subgroup of DLBCL patients.^303^ Somatic focal amplifications were identified that deregulated expression, resulting in significantly elevated *FCGR2B* levels that were associated with inferior response to rituximab‐containing immunochemotherapy.^303^ This study also observed increased frequencies of somatic SNVs and indels within *FCGR2B* introns, and suggested these alterations may result in a truncated protein by facilitating intron retention.^303^ Given the numerous preclinical studies^293^, ^296^, ^297^, ^304^ and smaller clinical trials^293^, ^294^ indicating high *FCGR2B* expression on tumor cells adversely affects rituximab‐based regimens, a series of large clinical cohorts were examined to investigate whether elevated *FCGR2B* levels in DLBCL imparts resistance to anti‐CD20 mAbs.^295^ Results demonstrated that high expression of *FCGR2B* serves as a robust, independent prognostic biomarker that is associated with shorter PFS in patients treated with rituximab immunochemotherapy, but not with obinutuzumab immunochemotherapy.^295^ Obinutuzumab does not internalize rapidly as it is a Type II anti‐CD20 mAb and is less impacted by FcγRIIb, supporting the earlier preclinical observations.^293^, ^297^ It was proposed, pending additional validation, that *FCGR2B* expression could be utilized to identify patients at an early stage who would likely derive the greatest benefit from obinutuzumab‐based treatment. Taken together, enhanced internalization capability—whether through the overexpression of the FcγRIIb2 isoform, or the ability of type I mAbs like rituximab to cluster and reorganize CD20 into lipid rafts—can diminish the availability of the mAb for its therapeutic action, potentially leading to poorer clinical outcomes. Of relevance, the absence of FcγRIIb‐mediated internalization of the type II mAb obinutuzumab was shown to enhance the phagocytosis of CLL target cells.^305^

It has also been shown that non‐hematopoietic tumors can ectopically express FcγRIIb, with one group demonstrating 40% of metastatic melanoma samples expressed the “B‐cell” FcγRIIb1 isoform.^306^, ^307^, ^308^ This suggests that ectopic expression of FcγRIIb may represent a progression factor that behaves as a decoy receptor to enable escape from FcγR‐dependent effector mechanisms in the presence of antitumor mAbs.^309^, ^310^ Utilizing murine in vitro and in vivo models, FcγRIIb1 was shown to enhance tumor formation and progression when ectopically expressed on nonlymphoid cells.^311^ Additionally, it has been shown that FcγRIIb expression is significantly increased on monocytes under high density,^312^ and on both monocytes and macrophages during hypoxia.^129^ Hypoxic conditions frequently occur in specific tissue niches, like bone marrow,^313^ and in poorly vascularized tumor microenvironments typical of advanced cancers. Hypoxia results in FcγRIIb upregulation in these areas, potentially limiting the effectiveness of direct targeting antibodies such as rituximab.^129^ Upregulation of *FCGR2B* has also been observed in macrophages that reside in the adipose tissue of breast cancer,^314^ as well as in macrophages and monocytes of colorectal‐, non‐small‐cell lung‐ and renal cell carcinomas with single‐cell RNA sequencing.^65^

Preclinical studies have demonstrated that high FcγRIIb expression within the tumor microenvironment also hinders the depletion of intratumoral Tregs by anti‐CD25 mAb, thereby reducing its effectiveness against established tumors.^315^ However, the therapeutic efficacy of anti‐CD25 mAbs can be restored by augmenting their binding to the activating FcγRs, through isotype switching to isotypes with higher A:I ratios, such as mIgG2a. Accordingly, anti‐CD25 mIgG2a overcame the high FcγRIIb expression in the TME to effectively deplete tumor‐infiltrating Tregs and enhance the control of established tumors.^315^

*FCGR2B* was also observed to be upregulated on a subset of highly differentiated, tumor‐infiltrating effector CD8+ T cells in both a melanoma mouse model and patients with metastatic melanoma.^316^ This upregulation was associated with an activated yet potentially exhausted CD8+ T‐cell phenotype and played a suppressive role in antitumor immunity. It was subsequently shown that *FCGR2B* mediated suppression of these CD8+ T cells, limiting the efficacy of mAb to both checkpoint inhibitors PD‐1 and CTLA‐4.^317^

## 10 REGULATION OF THE LOW‐AFFINITY *FCGR* LOCUS

Understanding how the genetic sequence, including variants, influences the regulation of *FCGR* expression has proven to be highly challenging, with limited research uncovering the underlying mechanisms.

Studies have demonstrated that genetic variants in the promoter and intronic regions of *FCGR2B* modulate TF binding and expression levels. A genetically determined down‐regulation of FcγRIIB1 in GCBs was linked to increased hyper‐IgG and IgG autoantibodies.^318^ Two substitutions and two deletion sites were identified within the promoter region that was thought to result in lower FcγRIIB1 expression and subsequently provided a mechanism by which B cells escape negative regulatory signals.^318^ Polymorphisms within the third intron were also identified which correlated with increased IgG antibody responses and are shared among autoimmune‐prone mouse strains.^319^, ^320^ Two unmethylated regions in the promoter and third intron have been characterized for their activities in the control of mFcγRIIb gene transcription in a cell type‐specific manner.^321^ These polymorphisms were reported to be involved in impairing the binding of the TF Activator protein 1 (AP‐1) and subsequently leading to enhanced germinal center formation and excessive autoantibody production,^320^, ^322^ and it was suggested similar alterations in *FCGR2B* regulatory regions could have implications in human autoimmune susceptibility.

The aforementioned upregulation of *FCGR2B* under hypoxic conditions,^323^, ^324^ which subsequently diminishes the FcγR A:I ratio and impairs phagocytosis of mAb‐opsonized targets was shown to be transcriptionally driven.^129^ TFs AP‐1 and members of the hypoxia‐inducible factor (HIF) family, HIF‐1α and HIF‐2α, were identified as critical upstream regulators of this increase in expression. Early research showed AP‐1 to bind a non‐canonical motif in the *FCGR2B* promoter region in cell lines,^325^ which was supported by a more recent study that utilized primary material to confirm binding at a non‐canonical AP‐1 motif 339‐bp upstream of *FCGR2B* TSS.^129^ Furthermore, nine hypoxia response elements (HREs) were identified within 15‐kb upstream of *FCGR2B* TSS, with the nearest canonical HRE motif at position −3916 upstream of TSS.^129^ During investigations of how to recalibrate the immunosuppressive FcyR signature of tumor‐associated macrophages, it was shown how activation of stimulator of interferon genes (STING) results in an increase in activating receptors FcyRIIa/FcyRIII while simultaneously decreasing expression of FcyRIIb.^326^ An equivalent increase in the A/I ratio in mice (increased FcyRIII/IV and decreased FcyRII) was also linked to a significant enhancement in mAb‐mediated depletion of malignant targets in the face of tumor‐mediated FcyR A/I suppression.^326^

Of the three polymorphic positions that make up the *FCGR3A*‐IH—which is overrepresented in individuals with elevated FcγRIIIa expression on NK cells and monocytes^106^—the 5′‐UTR ‐1405G>A SNP is reported to be situated within a ~200‐bp putative silencer region. It has been speculated that the SNP promotes increased expression by disrupting repressor binding to the silencer element.^106^ While further validation is required, luciferase reporter assays demonstrated deletion of this region resulted in more than a twofold increase in activity.^108^

One study has highlighted the potential role of FcγRIIa epigenetic regulation in KD; five key CpG sites within the *FCGR2A* promoter were associated with an increased risk of disease susceptibility and pathogenesis, with significant hypomethylation observed within the KD cohort compared with the control group.^327^ Furthermore, all five sites exhibited lower methylation levels in patients resistant to the intravenous immunoglobulin (IVIg) treatment relative to those who were responsive to IVIg.^327^ The KD cohort also demonstrated markedly elevated levels of *FCGR2A* mRNA, and reporter gene assays revealed that the five CpG sites were sufficient to regulate expression.^327^ A follow‐up study found *FCGR2A* expression levels were also significantly higher in IVIg‐resistant KD patients, and those with coronary artery lesions (CALs) compared with those without CALs.^328^

## 11 LIMITATIONS IN *FCGR* RESEARCH AND BENEFITS OF LONG‐RANGE GENOMICS

### 11.1 Current limitations

Investigation of the *FCGRs* has been hindered by the high intra‐region homology (Figure 2B–D), resulting in technical difficulties that lead to ambiguity and inconsistency of results. Genetic association studies involving *FCGR* variants are often unreliable due to technical variation, inconsistent nomenclature, high LD, and genetic complexity from segmental duplication.^329^, ^330^

As traditional methods for studying the *FCGRs*, including MLPA, Taqman genotyping, Haloplex, and whole exome/genome sequencing (WES/WGS), produce short units of data and heavily depend on the current human genome assembly, their capacity to characterize this highly homologous region is significantly restricted and can result in ambiguous data.^32^ Within the coding region of *FCGR2B*, there have been further nonsynonymous variants detected, but none have had their biological relevance, functional validation, or involvement in disease pathogenesis ascertained as yet.^8^ For instance, a stop codon within exon 3 (rs10917661) has been associated with SLE and ankylosing spondylitis^183^, ^331^ but due to the sequence homology, primers cannot distinguish between *FCGR2C* and *FCGR2B*. Therefore, it remains to be determined whether studies are reporting on a stop codon in *FCGR2B* or the already established Q57X variant within *FCGR2C*. Discrepancies in reports of the consequences of *FCGR2B* promoter haplotypes further illustrate these challenges.^125^ Due to genotyping of the low‐affinity *FCGR* locus being complex, it was noted that commonly used databases (such as NCBI BLAST and Ensembl) are not always accurate in the distinguishing between *FCGR* SNPs and genuine PSVs.^330^

Caution has also been recommended regarding ascertaining the true abundance of *FCGR* structural variants (SVs) when using the commonly used targeted techniques. For example, the frequency of *FCGR3B* CNV has previously been reported to range from 5% to 40% when using SYBR Green qPCR but with MLPA/PRT assays it has been defined as lower and much more consistent at 5%–8%.^121^, ^184^, ^192^, ^194^, ^196^, ^240^, ^332^ A meta‐analysis also reported that data across numerous studies that describe *FCGR3B* CNV were confounded by the type of experimental methodology that was utilized to assess copy number.^115^ However, one recent study compared qPCR, digital PCR (dPCR), and droplet digital PCR (ddPCR) for determining *FCGR3B* CNV and found complete concordance among the three platforms, including in rare cases with zero and four copies.^333^ While dPCR generally offers higher sensitivity and precision,^334^ this study concluded all three methods can quantify *FCGR3B* CN and claimed qPCR is still a viable option.^333^ Further highlighting the difficulties of accurately characterizing variation at the *FCGR* locus, an optimization and then evaluation of genomic analysis demonstrated that even MLPA and PRT assays produce discordant results when performed on the exact same cases with nearly 9% conflicting.^207^

These discrepancies then have serious consequences when interpreting findings in downstream analyses; for instance, qPCR has linked both low and high *FCGR3B* copy numbers with the incidence of SLE,^200^ while a triple PRT assay identified only the loss of *FCGR3B* as a contributing factor.^185^ Furthermore, with qPCR only, European SLE cohorts were documented to have a higher frequency of low *FCGR3B* copy number patients and no association was shown in different Chinese cohorts^196^, ^335^ and yet the PRT assay found a strong association with SLE in individuals from Hong Kong.^185^ Similarly, low *FCGR3B* has previously been associated with vasculitis^121^ but this has also been shown not to be the case when different groups used the same^196^ or different methodology.^184^ A recent study described a method that takes advantage of the high‐throughput data generated from whole‐genome aCGH.^114^ However, it was only able to distinguish heterozygous deletions that involved FCGR3A or FCGR3B with reasonable accuracy and thus could not differentiate between CNR2 and CNR3, or CNR1 and CNR4. An effort has also been made to use intensity values from an Immunochip platform assessing 18,039 individuals to define CNV. This study concluded it could identify 0, 1, and 2 copies but suspicion has been expressed on its reliability due to (i) not validating the findings with the “gold‐standard” MLPA/PRT techniques^329^ and (ii) not being able to demonstrate a relationship between *FCGR3B* CNV and FcγRIIIb expression (Nagelkerke et al. 2019a).^98^

One significant gap in knowledge within *FCGR* genomics is determining precise CNR breakpoints. Sequence homology confounds CNV analysis, making it notoriously difficult to ascertain whether the event is taking place on the proximal or distal repeat unit; impeding the accurate characterization of molecular events and their functional consequences.^159^ Many of the aforementioned studies utilize PSVs to differentiate the first paralogous block from the second—regardless of whether this is for assays involving qPCR, PRT, or MLPA. Relying on these PSVs means these technologies are limited due to their scarcity and ambiguity—that is, PSVs are infrequent and can often be polymorphic—leading to uncertainty in the origin of their products and thus impeding the precise localization of CNR boundaries.^34^, ^159^

*FCGR* analyses using high‐throughput short‐read sequencing are also significantly restricted as they face issues with multimapping. This issue is demonstrated in Figure 3 (light orange track) by the non‐uniform coverage of alignments for 148‐bp paired‐end genomic sequencing. The fact the four CNRs were only described in detail relatively recently is not too surprising considering that the novel chimeric events are undetectable with high‐throughput short‐read sequencing methods.^48^ The *FCGRs* high sequence identity prevents confident alignment thus WES is inadequate for detecting CNRs. It has also been speculated that unexplained cases of early onset severe autoimmunity may result from homozygous deletion of CNR4 because the *FCGR2B*‐stop variant would not be detected by NGS techniques as all *FCGR2B*‐stop reads would be mapped to the *FCGR2C* gene instead.^48^

In order to fully understand the clinico‐biological importance of the *FCGRs* and their variants in both normal and disease settings, it is crucial to have a high‐quality reference genome for any alignment‐based analyses. However, this is not the case for the *FCGR* locus and in the first ever human genome reference there was simply an empty gap where the *FCGR* genes reside.^336^ Moreover, the hg16 reference genome released in 2003 depicted only four of the low‐affinity *FCGR* genes in a region of just 90 kb, representing only one of the paralogous blocks. Since then, despite all the improvements to sequencing technologies, assembly algorithms, and updates to create the current GRCh38 reference genome, there are still deficiencies and uncertainties in accurate genome builds for the *FCGR* region.^32^ For example, the *FCGR2C*‐Q57X variant was initially annotated to be in the *FCGR2B* gene instead of *FCGR2C* in GRCh38. Following this, the bacterial artificial chromosomes (BACs) used to build the initial human reference have been sequenced with newer technologies and it has been determined that the paralogous region upstream of *FCGR2C* had been misassembled and swapped with the homologous region on *FCGR2B*.^303^

Some of the greatest technical challenges with short‐read sequencing technologies are caused by repetitive DNA: sequences that are identical or similar to other regions in the genome, including segmental duplications.^337^, ^338^ Accordingly, it has been demonstrated that more than 70% of structural variation in the human genome could be undetectable with reads less than 300 bp in length.^339^ Consequently in highly homologous regions like the *FCGR* region where the two paralogous blocks have been reported to share >95% homology,^35^ issues are encountered when trying to accurately align or assemble the reads. The repetitive nature creates technical challenges for the alignment and assembly software, leading to difficulties in conclusively resolving which genomic location a read originated from and introduces ambiguities from a computational perspective in the form of biases and errors when interpreting the results. As demonstrated in Figure 3 (black and orange tracks), short reads often have uneven coverage, regions where reads do not align, or do not map uniquely or with high quality. This causes issues with downstream analysis, especially when trying to study regulation with techniques that utilize very short reads, such as the 25‐75‐bp read length that is common in ChIP or ATAC‐seq studies. However, the rise of third‐generation sequencing technologies that produce long reads may offer solutions to these problems, potentially advancing our understanding of the genomic structure and regulation of the *FCGRs*.

#### 11.1.1 The potential benefits of increased sequence read length

For more than a decade, high‐throughput short‐read sequencing has dominated genomics research with wide‐ranging applications, enriching our understanding of evolution and disease through the study of SNPs, CNV, and indels.^340^, ^341^, ^342^ Its high throughput has also facilitated the study of a multitude of other biological phenomena, including gene expression, chromatin accessibility, and TF binding. Yet regardless of the ability to produce highly accurate (>99.9%) sequencing reads, the sequence‐by‐synthesis process results in a limited read length of only 50–300 bp,^343^ leading to the issues described above. Therefore, alternative, complementary methods, mainly involving long‐read approaches such as nanopore‐ and single‐molecule real‐time (SMRT) sequencing‐based technologies, have been developed.

Nanopore sequencing generates reads that typically range from one to >100 kilobases (kb)^344^, ^345^, ^346^ and excels in producing ultra‐long reads, achieving up to 4.1 megabases (mb).^347^ SMRT sequencing approaches can generate two different types of long‐read data that differ in both read length (5–60 kb) and accuracy (ranging from 87% to >99%).^348^ Optical genome mapping (OGM) is an additional long‐range technology that can aid in detecting SVs. It generates 50–100‐mb genomic maps by de novo assembly of fluorescently labeled DNA molecules. Comprehensive SV analysis can be performed by inputting these molecules into haplotype‐aware assemblers and comparing the final consensus maps to the reference genome. However, unlike sequencing platforms, optical mapping cannot provide bp resolution and is limited to detecting SVs of at least 500 bp in length.^349^

Human genetics research has already benefited from these advances in long‐read sequencing, improving genome assembly, variant discovery, disease association, and population genomics.^350^, ^351^, ^352^, ^353^ It has provided tools with the capacity to resolve some of the most complex, challenging, and dynamic regions of the human genome thus dramatically improving the understanding of human heritability, diversity, mutational mechanisms, and the genetic basis of disease.

In addition to the significant improvement to individual read lengths, long‐read sequencing can also result in a more even distribution of coverage across the genome as it is not as sensitive to imbalanced sequence composition—unlike short‐read platforms which often leave regions of high GC content with low or no read depth.^354^ A systematic analysis showed that over 15mb of the human genome (from >35,000 different regions) are left in the “dark” when only using short‐read sequencing, hence preventing researchers from detecting disease‐relevant polymorphisms.^355^ These dark regions, where short‐read data cannot be adequately aligned or assembled, can either be “dark by depth” with no or few mappable reads or “dark by mapping quality.” Sequences that are inherently challenging to sequence at the molecular level, like those with high GC content, give rise to dark by depth regions. In contrast, dark by mapping quality regions arise due to bioinformatic difficulties and ambiguities. This is particularly true of duplicated regions where mapping a short‐read to a unique position is impossible due to insufficient confidence, thereby creating “camouflaged” regions. Consequently, long‐read technologies have begun to resolve the sequence of difficult‐to‐analyze areas. A capture panel targeting medically relevant dark genomic regions for SMRT sequencing has been generated.^356^ While it encompasses nearly 400 complete genes, only *FCGR1A*, *FCGR2B*, and *FCGR3A* are included with respect to the *FCGR* genes.

Now that read lengths are of a sufficient size to traverse the most complex and repetitive genomic structures, long‐read sequencing has also improved the de novo assembly of genomes, achieving greater contiguity compared with short‐read and Sanger‐based methods.^348^ Moreover, combining complementary datasets generated with orthogonal technologies provides superior genome assembly. For example, the telomere‐to‐telomere (T2T) consortium utilized different long‐ and short‐read technologies in their effort to construct the very first, truly complete assembly of a human genome. The T2T project has so far released the complete X chromosome,^345^ closed the gaps of the first autosome chromosome 8,^357^ and presented the entire 3.06 billion bp sequence of all human chromosomes (except Y): resolving complex regions, improving variant calling accuracy, and introducing nearly 200 million more bp of sequence to be explored.^358^ Building on the success of the T2T project, the Human Pangenome Reference Consortium (HPRC) was launched with the objective to construct complete T2T reference genomes for every population, striving to promote equitable healthcare for every patient.^359^ This initiative is striving to address the lack of global representation in the current human reference genome—which despite being the most widely utilized resource in human genetics, is comprised of collapsed haplotypes from primarily a single individual. By employing a wide range of platforms, the HPRC aims to create high‐quality, contiguous diploid genomes that represent the entire scope of diverse human variation.^360^

Long‐read sequencing has also advanced the phasing of genomic sequences, revealing insights into both maternal and paternal haplotypes. This enhances accurate variant detection and is particularly valuable for resolving SV, as differences between haplotypes in short‐read analyses can often result in collapsed or hybrid representations that do not reflect true haplotype architecture.^361^, ^362^ Long‐read technology enables the assembly of complete haplotypes and improves our understanding of the comprehensive spectrum of genomic variants.^363^, ^364^, ^365^ For example, one study that implemented a multiplatform approach on the same individuals revealed that 46% of deletions and 78% of insertions went undetected by short‐read WGS, despite employing 11 different variant‐calling software tools.^339^ Furthermore, despite impacting the greatest proportion of nucleotides within the genome than any other category of sequence variant, SVs have typically been understudied compared with SNVs.^342^, ^366^, ^367^ It has previously been reported that SV detection approaches using short reads perform poorly in some regions, demonstrating low sensitivity,^364^, ^368^ often misinterpreting complex SVs^369^ and exhibiting high rates of false positives.^366^, ^370^, ^371^ Notably, the homology and complexity of the *FCGR* region have confounded accurate SV annotation, with long‐read technologies now making important progress as discussed below.

A particular strength of nanopore sequencing is its ability to analyze all nucleotides, including modified DNA and RNA bases. This allows for the detection of epigenetic modifications, with reports thus far of base‐callers trained to detect 4‐methylcytosine, 5‐methylcytosine, 5‐hydroxymethylcytosine, N6‐methyladenine, and 8‐oxoguanine.^372^, ^373^, ^374^, ^375^, ^376^, ^377^ In addition to aiding in the study of allelic‐specific epigenetic variation, it has been used to study the distinctive epigenomic landscape and differential methylation of structurally variant regions associated with cancer.^378^

#### 11.1.2 The application of long‐read technologies to the *FCGR* locus

Long‐read sequencing now facilitates more accurate phased assemblies, enhanced mapping quality, and reduced bioinformatic uncertainties for the study of the *FCGR* locus.^379^ As discussed above, previous analysis of the *FCGR* locus was significantly limited by short reads and alignment‐dependent methods, particularly due to the issues with the existing human reference genomes when assembling segmental duplications such as the *FCGR* region with short (<1000 bp) reads. However, while long‐read technologies are overcoming many of these issues aiding advanced variant detection and disease understanding, this has often only occurred subsequent to a high‐quality assembly of the region of interest.^348^

To date, a comprehensive characterization of the low‐affinity *FCGR* locus that accurately depicts all variants has not been published. While four CNRs have been identified, short‐read methods have not precisely resolved their boundaries or CNV‐generating breakpoints. Moreover, it is known large segmental duplications encourage additional unequal cross‐overs between highly homologous sequences via further NAHR events^380^, ^381^, ^382^; thus, novel CNV could exist that earlier short‐read, unphased approaches could not detect. The same limitations, regarding previously unexplored variants and their impact, also apply to *FCGR* SNPs—particularly those that are involved in expression regulation as nonsynonymous SNPs within exons have historically been the primary focus. For example, a 2018 study into the full‐length of *FCGR3A* with nanopore sequencing revealed it is more polymorphic than the literature had previously described with many novel variants residing in noncoding regions.^383^ As shown in Figure 3, long‐read technologies are now beginning to overcome many of these issues, increasing coverage, depth, and resolution.

## 12 SUMMARY AND FUTURE DIRECTIONS

In summary, the FcγRs are crucial regulators of a well‐coordinated and effective immune response, influencing pathogen defense, susceptibility to autoimmunity, and the success of mAb therapies. Encoded by highly homologous genes shaped by evolutionary duplication events, the *FCGR*s exhibit significant genetic variation, including SNPs and SVs, which can impact their affinity, expression, function, and clinical relevance. The extensive polymorphic nature of the *FCGR* loci coupled with the high sequence similarity presents challenges in analysis. While some associations between genetic variants and disease have been identified, further investigation is necessary to address existing difficulties and confounding LD. Advancements in long‐read sequencing offer opportunities to overcome these challenges, providing a more comprehensive understanding of the genetic sequence, variants, regulation, and role in disease.

Due to the limitations of previously used technology, some caution should be exercised when examining historical genetic studies. Careful consideration is needed as much of the research does not account for the full spectrum of genetic variants or have adequately stratified controlled groups, potentially leading to incomplete or misleading conclusions. To accurately identify causative variants at the *FCGR* locus, comprehensive genotyping of all functional genetic variants is important. The necessity for accurate variant identification has led to multiple revisions of the MLPA assay since its introduction, allowing for the determination of independent risk markers through multiple logistic regression analysis.^98^, ^330^ However, the challenges in genotyping this locus mean that some functionally relevant variants may still remain undetected and thus focus should not be limited to nonsynonymous SNPs as all variants, including those in noncoding regions, need to be considered as they can play a critical role in gene regulation, expression, and splicing. It has also been emphasized that selecting population‐matched control groups in genetic studies is vital for accurate association analyses^98^ as incongruence between control and patient populations can confound findings and obscure the true genetic contributions to disease.

The strong LD in the region also complicates efforts to determine which genetic variants contribute to specific traits or diseases. To effectively deconvolute these effects, studies should leverage large, well‐characterized cohorts. With larger sample sizes, it becomes possible to assess the influence of different variant combinations, helping to clarify their individual roles in disease risk or therapeutic outcomes. Given the strong LD, it could be more informative to report some of *FCGR* genetic variation as haplotypes rather than as individual variants, similar to the established approach used for the *FCGR3B*‐HNA SNPs.^98^ Future research also requires large cohorts to gain a comprehensive understanding of *FCGR* variation, including accurately distinguishing between paralogous and allelic variation, and uncovering any additional CNRs that have gone undiscovered.

The current genome reference sequence (GRCh38) is limited by its reliance on a small number of individuals, with around 70% of the sequence derived from just one donor.^384^, ^385^ This patchwork approach of collapsed haplotypes introduces biases and inaccuracies, particularly in regions that are difficult to assemble like the *FCGR* locus, and may not reflect the diversity of the general population, consequently impacting the reliability of variant identification and association with disease.^386^, ^387^, ^388^ De novo assembly of the *FCGR*s across diverse populations promises to create more representative and accurate genomic references that will enhance the detection of novel variants and improve the precision of genetic studies by providing a more comprehensive view of population diversity and variation.

Expansive datasets, such as those provided by national and international Biobanks, are invaluable for unraveling the complexities of genetic contributions to phenotypes. These large‐scale resources not only facilitate the identification of novel genetic variants but also allow for a robust evaluation of previous associations, either confirming or challenging earlier findings with greater precision. Hujoel et al.'s analysis of the UK Biobank's extensive dataset, which includes WES for 470,000 participants, utilized haplotype‐informed CNV detection to link *FCGR3B* CNV with COPD susceptibility.^122^ This approach underscores the importance of comprehensive large‐scale datasets in advancing our grasp of complex phenotypes and translating *FCGR* genetic findings into practical clinical applications.

In conclusion, to fully understand the genetic variation at the *FCGR* locus, future research must undertake exhaustive analyses of all relevant SNPs, CNRs, and their haplotypes. Such comprehensive studies are crucial for deciphering the complexities of disease associations and optimizing therapeutic strategies, especially in immunotherapy. Achieving these goals will depend on large, well‐characterized cohorts and diverse control groups alongside technology capable of long‐range analysis such as long‐read sequencing. Progress in understanding FcγR genetics could readily advance personalized medicine and we hope to see the development of more effective treatments tailored to individual genetic profiles in the future.

## FUNDING INFORMATION

Funding was provided by Cancer Research UK program grants (award numbers: A24721; DRCRPG‐May23/100001) and Cancer Research UK and Experimental Cancer Centre awards C328/A25139 and C24563/A25171.

## CONFLICT OF INTEREST STATEMENT

M.S.C. acts as a consultant for a number of biotech companies, being retained as a consultant for BioInvent International, and has received research funding from BioInvent, GSK, UCB, iTeos, and Roche.

## Data Availability

Data sharing not applicable—no new data is generated.
